# Supplementary material for: Germanium disulfide as an alternative high refractive index and transparent material for UV-visible nanophotonics
Source: Light Sci Appl. 2025 Jun 18;14:213. doi: 10.1038/s41377-025-01886-y (PMC12174367; doi:10.1038/s41377-025-01886-y)
Supplement: Supplementary file 1 — Supplementary Information [file 41377_2025_1886_MOESM1_ESM.pdf]

Supplementary Information for

Germanium Disulfide as an Alternative High Refractive Index and Transparent Material for UV-Visible Nanophotonics

Aleksandr S. Slavich[1†], Georgy A. Ermolaev[1†], Ilya A. Zavidovskiy[2†], Dmitriy V. Grudinin[1†], Konstantin V. Kravtsov[1†], Mikhail K. Tatmyshevskiy[2], Mikhail S. Mironov[1], Adilet N. Toksumakov[1], Gleb I. Tselikov[1], Ilia M. Fradkin[1], Kirill V. Voronin[3], Maksim R. Povolotskiy[2], Olga G. Matveeva[2], Alexander V. Syuy[1], Dmitry I. Yakubovsky[2], Dmitry M. Tsymbarenko[4], Ivan Kruglov[1], Davit A. Ghazaryan[2,5], Sergey M. Novikov[2], Andrey A. Vyshnevyy[1], Aleksey V. Arsenin[1], Valentyn S. Volkov[1] and Kostya S. Novoselov[6,7,8*]*

[1]Emerging Technologies Research Center, XPANCEO, Internet City, Emmay Tower, Dubai, United Arab Emirates

[2]Moscow Center for Advanced Studies, Kulakova str. 20, Moscow, 123592, Russia

[3]Donostia International Physics Center (DIPC), Donostia/San Sebastián, 20018, Spain

[4]Department of Chemistry, Lomonosov Moscow State University, Moscow, 119991, Russia

[5]Laboratory of Advanced Functional Materials, Yerevan State University, Yerevan 0025, Armenia

[6]National Graphene Institute (NGI), University of Manchester, Manchester, M13 9PL, UK

[7]Department of Materials Science and Engineering, National University of Singapore, Singapore, 03-09 EA, Singapore

[8]Institute for Functional Intelligent Materials, National University of Singapore, 117544, Singapore, Singapore

[†]These authors contributed equally to this work

*Correspondence should be addressed to e-mail: kostya@nus.edu.sg and vsv@xpanceo.com

Table of Contents

Supplementary Note 1: Crystallographic characterization of GeS_2

Supplementary Note 2: First-principle calculations

Supplementary Note 3: Angle-resolved Raman spectroscopy

Supplementary Note 4: Ellipsometry analysis for determination of GeS_2 optical constants

Supplementary Note 5: Processing of polarization-dependent transmittance spectra for GeS_2

Supplementary Note 6: Scanning near-field optical microscopy of GeS_2

Supplementary Note 7: Tabulated optical constants of GeS_2

Supplementary Note 8: Polarized transmittance calculations (transfer matrix method)

Supplementary Note 1: Crystallographic characterization of GeS_2

In this note, we present detailed information on the X-ray diffraction (XRD) analysis performed to elucidate the crystalline structure of GeS_2 bulk crystal (see Figure S1). Details of the data collection and refinement parameters are summarized in Table S1. Unit cell content, thermal ellipsoids and atoms numeration scheme are depicted in Figure S2. Selected interatomic distances and bond angles are listed in Table S2. It is worth noting that the crystal structure of monoclinic GeS_2 was previously reported by [1] with the resulting R-factor of 9%. By applying absorption correction in our study, we have achieved a more accurate crystal structure determination with a significantly lower R-factor (3.6%), thereby enhancing the precision of the unit cell parameters and atomic positions (Table S3).

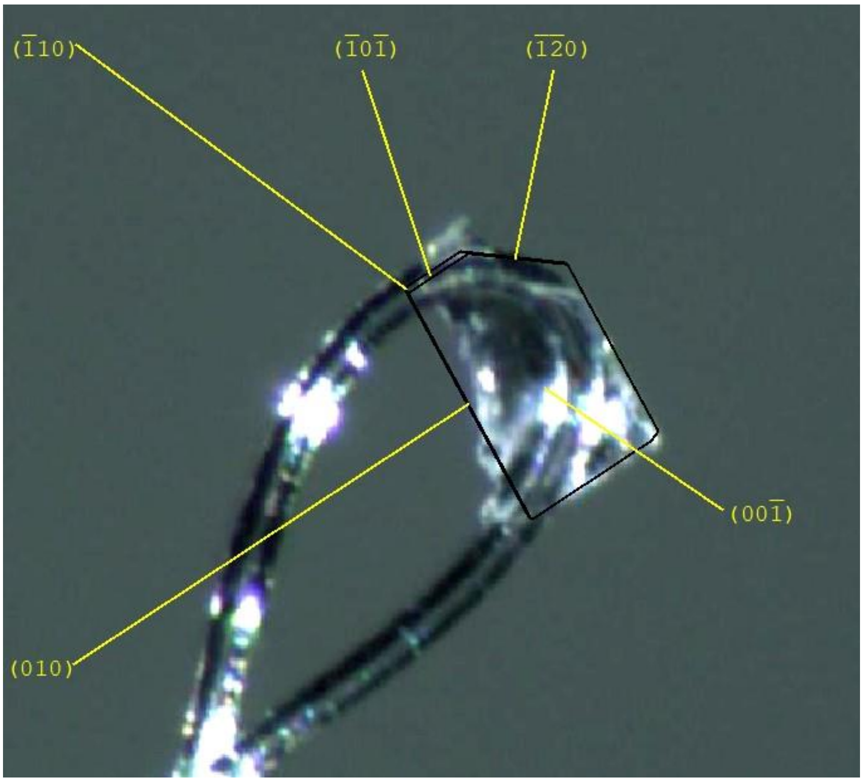

Figure S1. Habit of the GeS_2 single crystal mounted on the cryoloop. Indexed crystal faces are depicted by black lines. Yellow lines show the face normal with the respective Miller indexes. Crystal has plate shape with the dimensions of 0.006×0.110×0.267 mm^3.

Table S1. Summary of the crystal structure and refinement details for GeS_2.

Formula	GeS_2
Formula weight (g·mol^{-1})	136.71
Diffractometer	Bruker D8 QUEST
Radiation, wavelength (Å)	Mo Kα, 0.71073
Data collection method	ω scans
Temperature (K)	300(2)
Crystal system	Monoclinic
Space group	$P2_1/c$
a (Å)	6.6946(14)
b (Å)	16.037(3)
c (Å)	11.423(2)
α (°)	90
β (°)	90.935(7)
γ (°)	90
V (Å^3)	1226.2(4)
Z	16
Colour, habit	colorless, plate
Crystal dimensions (mm)	0.006×0.110×0.267
Density D_{calc} (g·cm^{-3})	2.962
μ (mm^{-1})	11.018
Unique reflections (R_{int})	2686(0.0703)
Observed reflections [$I > 2\sigma(I)$]	2082
Parameters	109
$R_1[I > 2\sigma(I)]$, ωR_2	0.0358, 0.0921
Goodness of fit on F^2	0.965
Absorption correction	numerical
T_{min}, T_{max}	0.1131, 1.0000
ρ_{min}, ρ_{max} (eÅ^{-3})	−0.683, 1.142

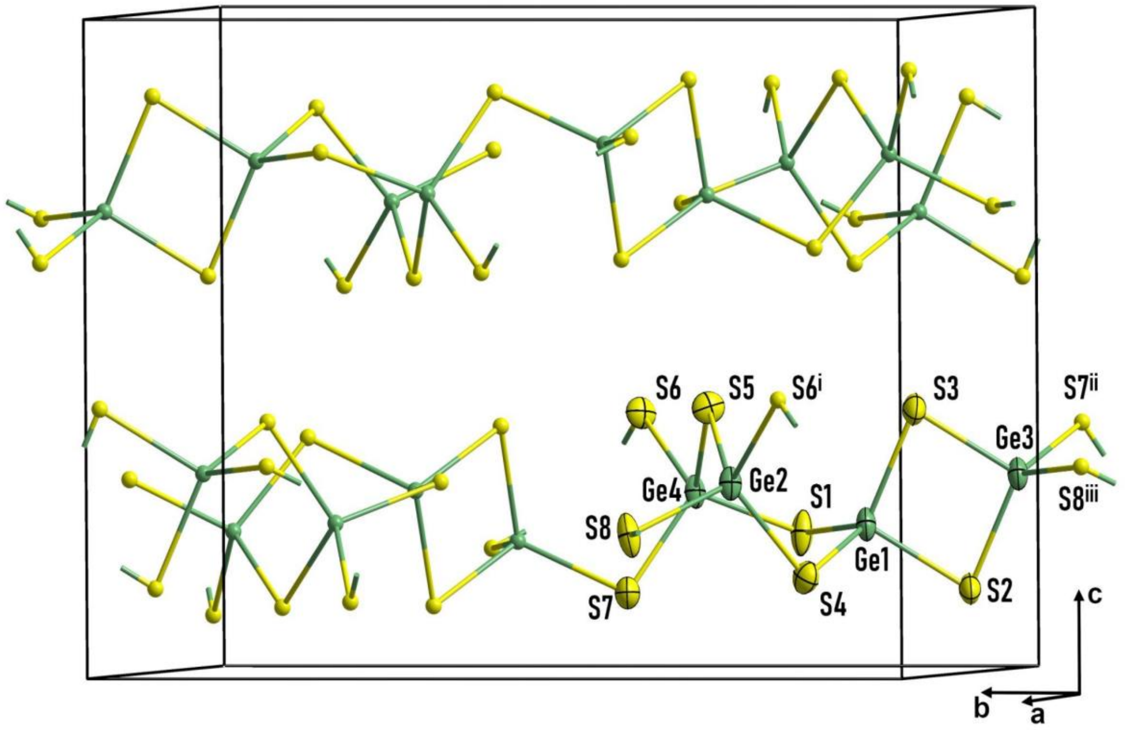

Figure S2. Fragment of crystal structure of GeS_2. Atoms within the asymmetric part of unit cell are depicted as thermal ellipsoids with 50% probability. Symmetry codes: (i) *x – 1, y, z*; (ii) *1 – x, y – 0.5, 0.5 – z*; (iii) *– x, y – 0.5, 0.5 – z*.

Table S2. Interatomic distances (Å) and bond angles (°) in GeS_2 structure. Symmetry codes: (i) *x – 1, y, z*; (ii) *1 – x, y – 0.5, 0.5 – z*; (iii) *– x, y – 0.5, 0.5 – z*; (iv) *1 – x, 0.5 + y, 0.5 – z*.

Parameter	Distance	Parameter	Angle
Ge1−S1	2.2138(14)	S1−Ge1−S2	108.24(5)
Ge1−S2	2.2160(14)	S1−Ge1−S4	110.69(5)
Ge1−S3	2.2212(15)	S1−Ge1−S3	115.79(6)
Ge1−S4	2.2191(14)	S2−Ge1−S3	98.01(5)
Ge2−S4	2.1983(14)	S2−Ge1−S4	108.12(6)
Ge2−S5	2.1967(14)	S4−Ge1−S3	114.80(5)
Ge2−$S6^{i}$	2.2102(13)	S4−Ge2−$S6^{i}$	113.35(5)
Ge2−S8	2.2113(14)	S4−Ge2−S8	108.24(6)
Ge3−S2	2.2243(14)	S5−Ge2−S4	111.64(5)
Ge3−S3	2.2126(14)	S5−Ge2−$S6^{i}$	100.50(5)
Ge3−$S7^{ii}$	2.2270(14)	S5−Ge2−S8	111.91(5)
Ge3−$S8^{iii}$	2.2124(14)	$S6^{i}$−Ge2−S8	111.13(5)
Ge4−S1	2.2057(14)	S2−Ge3−$S7^{ii}$	114.37(5)

Ge4–S5	2.2152(13)	S3–Ge3–S2	98.02(5)
Ge4–S6	2.2013(14)	S3–Ge3–S7ii	109.41(6)
Ge4–S7	2.2034(15)	S8iii–Ge3–S2	115.59(6)
Ge1···Ge3	2.9103(9)	S8iii–Ge3–S3	108.72(5)
Ge1···Ge2	3.4064(9)	S8iii–Ge3–S7ii	109.88(5)
Ge1···Ge4	3.3735(8)	S1–Ge4–S5	111.37(5)
Ge2···Ge4	3.4277(9)	S6–Ge4–S1	112.56(5)
Ge4···Ge3iv	3.3489(9)	S6–Ge4–S5	96.94(5)
		S6–Ge4–S7	113.00(5)
		S7–Ge4–S1	108.37(6)
		S7–Ge4–S5	114.36(5)

Table S3. Unit cell parameters of monoclinic GeS_2.

Parameter	This work	Ref.[1]	Parameter
a (Å)	6.6946(14)	6.720(3)	*a* (Å)
b (Å)	16.037(3)	16.101(3)	*b* (Å)
c (Å)	11.423(2)	11.436(3)	*c* (Å)

To further verify the crystal structure of GeS_2 flakes, we performed select-area electron diffraction (SAED) measurements. An optical image of as-transferred GeS_2 onto TEM grid and the related SAED pattern are presented in Figure S3a,b. In Figure S3c, the SAED pattern is overlaid with a calculated pattern derived from XRD crystallographic data, demonstrating a precise match and indicating a good corroboration between the experimental and calculated patterns. To confirm the material quality of the studied GeS_2 flakes we determine the relative ratio of the element's concentration by energy-dispersive X-ray spectroscopy (EDS). In quantitative analysis, the concentration of a specific element present in fakes was measured by the analysis of peaks in the EDS spectrum. EDS results in Figure S3d demonstrate that the presence of 35.9 at.% germanium and 64.1 at.% sulfur experimentally confirmed the stoichiometric ratio of GeS_2 with high accuracy.

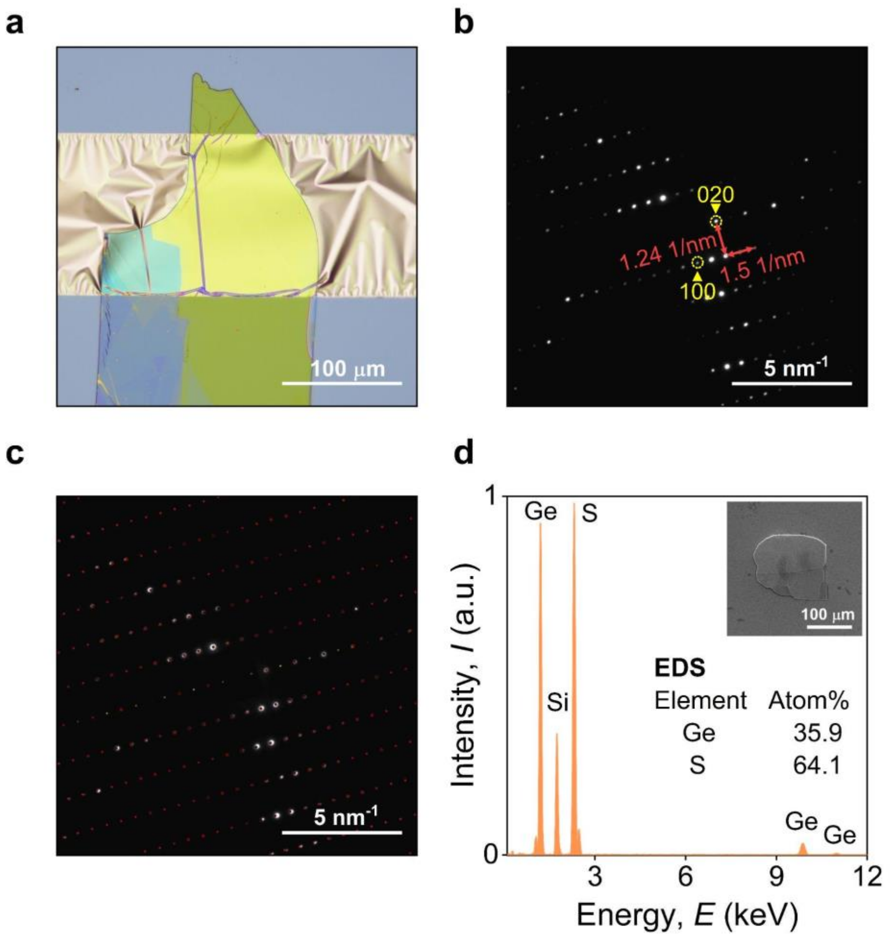

Figure S3. GeS_2 TEM characterization. **(a)** Optical image of mechanically exfoliated GeS_2 sheet transferred over TEM membrane. **(b)** Experimental selected area electron diffraction pattern across ac crystallographic plane. **(c)** TEM simulation pattern (color dots) on top of the experimental micrograph presented in **(a)**. **(d)** EDS characterization of the GeS_2 flake transferred on Si wafer. The inset shows SEM image of the as-transferred GeS_2 flake for EDS analysis.

Supplementary Note 2: First-principle calculations

For all the first-principle calculations the lattice parameters as well as atomic positions of GeS_2 were taken from our XRD results (Supplementary Note 1). We calculated static dielectric tensors for experimental unit cells of all materials presented in Figure 1d (MoS_2 ICSD file: 49801; WS_2 ICSD file: 202366; SnS_2 ICSD file: 100610; GaS ICSD file: 59; As_2S_3 CSD file: 2258216; MoO_3 ICSD file: 35076; $GeSe_2$ ICSD file: 614; hBN ICSD file: 241785), based on the density-functional-perturbation theory (DFPT) method implemented in VASP (Vienna Ab initio Simulation Package). For this kind of computation, we used a plane wave basis energy cutoff of 550 eV and Γ-centered k-point grid to sample the Brillouin zone at a resolution of $2\pi\times0.03$ Å^{-1} for all the materials. We used pseudopotentials recommended on the VASP website.

Lattice dynamics calculations of GeS_2 were performed using the Phonopy code[2,3], where VASP was used to calculate interatomic forces[4]. Calculations were performed under Perdew-Burke-Ernzerhof (PBE) exchange-correlation functional along with Grimme-D3 scheme to account for van der Waals interactions[5,6]. For modeling ionic cores, the projector augmented wave (PAW) pseudopotentials were used treating the Ge 3d, 4s and 4p and the S 3s and 3p electrons as valence[7,8]. A kinetic energy

cutoff for the plane wave basis set was set to 450 eV. k-meshes of 7×3×4 and 3×2×2 subdivisions were used for the GeS_2 primitive cell and supercells, respectively. The second-order force constants were calculated for 2×1×2 supercell (192 atoms) with a finite displacement step of 0.01 Å. A tolerance of 10^{-8} eV was set for total-energy calculations. We also considered a non-analytical term correction (NAC) for a dynamical matrix to account for long-range dipole-dipole interactions[9–11].

Intensities of Raman peaks were calculated under PBE functional using the VASP package. For a static dielectric matrix and Born effective charges calculations, a DFPT was used as implemented in VASP in the case of PBE. Linewidths were chosen to be 2 cm^{-1}. A space group of GeS_2 is $P2_1/c$ (monoclinic system), therefore Raman-active modes are A_g and B_g. In the current experimental setup, all the active modes are observed. Therefore, we performed Phonopy calculations for all modes with A_g and B_g irreducible representations at Γ-point.

Optical properties of GeS_2 were calculated within PBE[5], HSE06[12], PBE+GW[13,14] and HSE06+GW[15] approximations using the VASP code. In the GW approach, macro- and micro-dielectric tensors were also calculated. The comparison of different methods is shown in Figure S4. The best match between experiment and theory (Figure S4e) was for GW+PBE approach and macro-dielectric tensor for in-plane and out-of-plane optical properties.

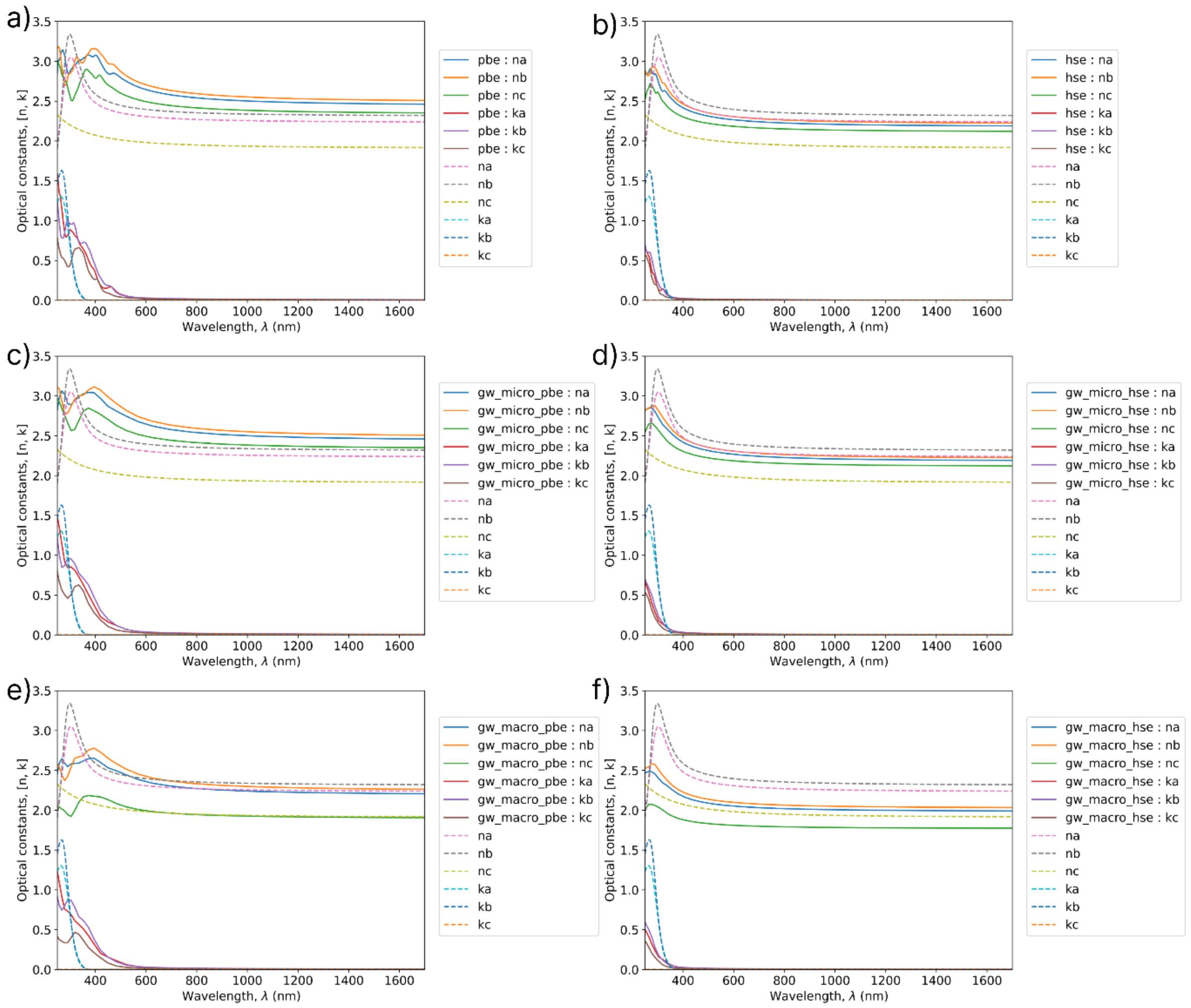

Figure S4. Optical properties of GeS_2 calculated within **(a)** PBE, **(b)** HSE06, **(c)** GW+PBE (from micro dielectric tensor), **(d)** GW+HSE06 (from micro dielectric tensor), e) GW+PBE (from macro dielectric tensor), **(f)** GW+HSE06 (from macro dielectric tensor). Theoretically calculated data for refractive index is shown with solid lines, experimental data - with dotted lines and points.

Supplementary Note 3: Angle-resolved Raman spectroscopy

In the current study, analysis of the materials' vibrational response was carried out by polarized Raman studies combined with density functional theory (DFT) calculations of Brillouin-zone center phonons. For GeS_2 with $P2_1/c$ space group, irreducible representation of Γ point phonons are the following[16]:

$$\Gamma = \Gamma_{\text{acoustic}} + \Gamma_{\text{optic}}$$

$$\Gamma_{\text{acoustic}} = A_u + 2B_u$$

$$\Gamma_{\text{optic}} = 36A_g + 35A_u + 36B_g + 34B_u$$

Among these phonons, A_g and B_g are Raman-active. In the current experimental setup, the linearly polarized beam is parallel to the interlayer direction. As shown in Figure S5, spectra measured with incident light polarization parallel to analyzer ($\boldsymbol{e_s} \,//\, \boldsymbol{e_L}$) and without analyzer (*unpolarized*) are relatively similar, while Raman lines intensity in cross-polarization ($\boldsymbol{e_s} \perp \boldsymbol{e_L}$) spectrum is negligible in comparison to the one measured at $\boldsymbol{e_s} \,//\, \boldsymbol{e_L}$. This is also qualitatively confirmed by ab initio comparison of $\boldsymbol{e_s} \,//\, \boldsymbol{e_L}$ and $\boldsymbol{e_s} \perp \boldsymbol{e_L}$ angle-averaged spectra (see Figures S5,6). This assessment allows us to ensure that the intensity of $\boldsymbol{e_s} \,//\, \boldsymbol{e_L}$ components of A_g and B_g modes significantly exceeds the one of $\boldsymbol{e_s} \perp \boldsymbol{e_L}$ components of A_g and B_g modes, and to conclude that parallel-polarized theoretical components represent experimental spectra with a sufficient accuracy. Taking this into account, for the analysis of the angle-resolved Raman spectra we used a parallel configuration of the analyzer, which allowed us to carry out the ab initio analysis for $\boldsymbol{e_s} \,//\, \boldsymbol{e_L}$ components of A_g and B_g modes only.

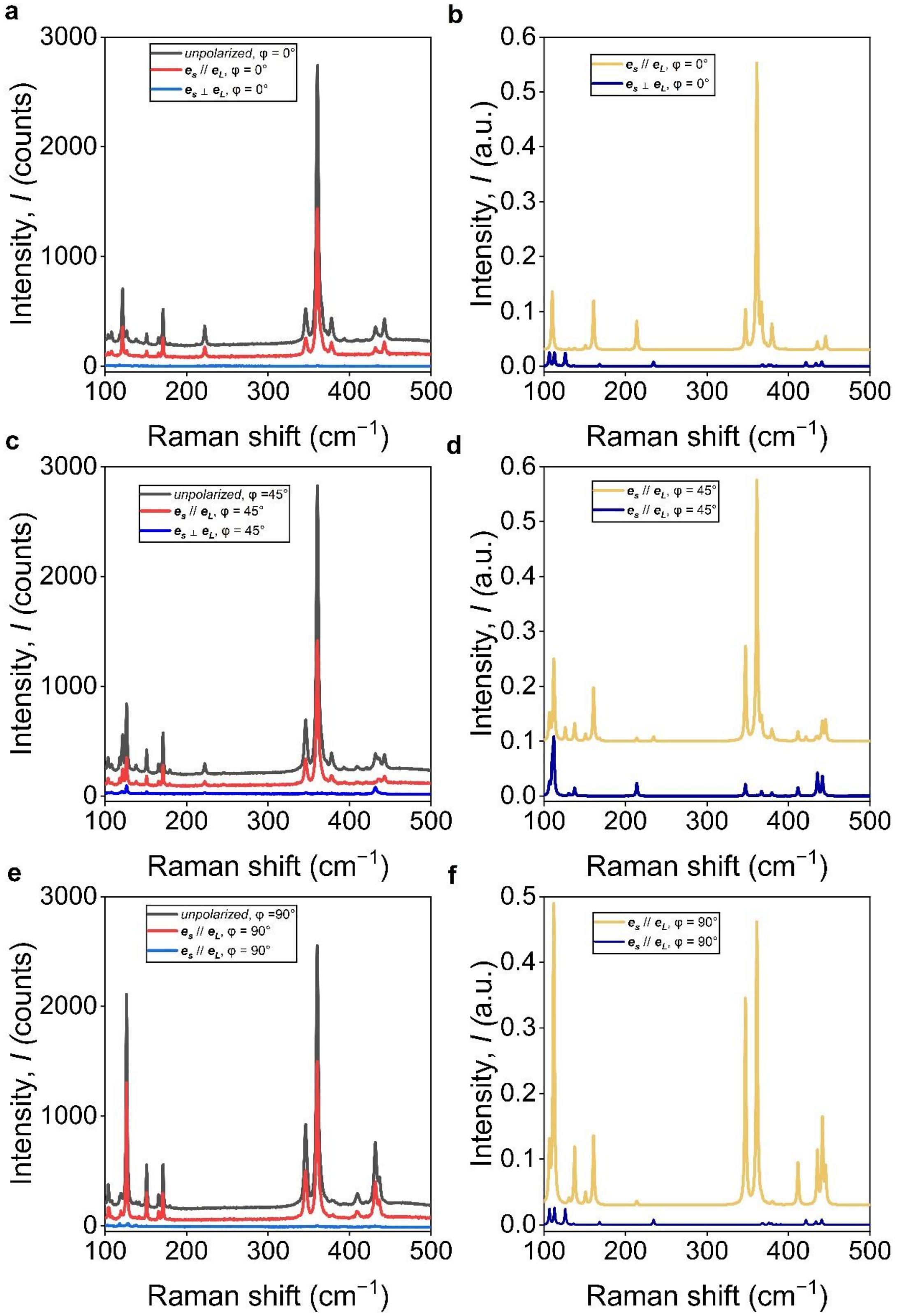

Figure S5. Comparison of experimental **(a, c, e)** and theoretical **(b, d, f)** Raman spectra measured and modeled at various angles ϕ of sample rotation (**a,b**: φ = 0°, **c,d**: φ = 45°, **e,f**: φ = 90°). Angles are measured from the crystallographic axis *a*. Spectra were measured with optical analyzer with the transmission axis directed perpendicular to the polarization of the laser beam ($\boldsymbol{e_s} \perp \boldsymbol{e_L}$, blue curve); (2) with optical analyzer with the transmission axis directed parallel to the polarization of the laser beam ($\boldsymbol{e_s}$ // $\boldsymbol{e_L}$, red curve); (3) without analyzer (*unpolarized*, black curve). Vertical offset is applied to distinguish the lines.

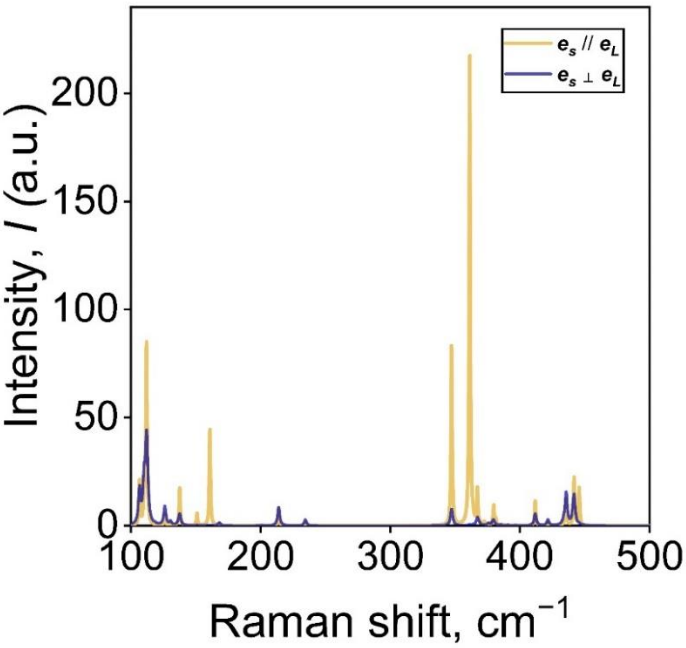

Figure S6. First principles non-normalized angle-averaged spectra for cross (blue) and parallel (orange) cases (polarization vectors lie in *ab*-plane).

For GeS_2, both A_g and B_g modes contribute to the spectra. DFT calculations, which results are shown in Figure S7, prove that B_g modes are considerably less intensive. Our theoretical computations show that the most intense B_g peak is about 40 times less intense than 360-cm^{-1}-centered A_g^6 peak in the angle-averaged parallel configuration.

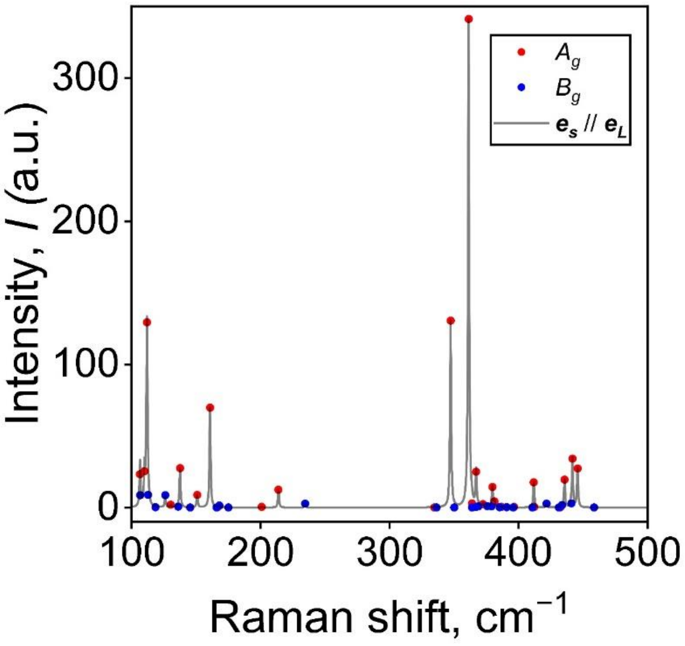

Figure S7. DFT angle-averaged intensities of all Raman-active modes above 100 cm^{-1} versus Raman shift in parallel configuration. Calculated angle-averaged spectrum for parallel $\boldsymbol{e_s}$ // $\boldsymbol{e_L}$ configuration is shown as a reference.

For angle-resolved Raman spectroscopy, the A_g/B_g mode type determines the type of the polar plot. More specifically, the intensity of the vibrational mode is proportional to $|\boldsymbol{e_s} \times \hat{R} \times \boldsymbol{e_L}|^2$, where $\hat{R}$ is a Raman tensor of the particular mode, $\boldsymbol{e_s}$ and $\boldsymbol{e_L}$ are polarization vectors of scattered light and laser beam respectively. For A_g and B_g modes of monoclinic lattices with *b* being a unique axis, Raman tensors are represented by the following expressions:

$$\hat{R}(A_g) = \begin{pmatrix} a & 0 & d \\ 0 & b & 0 \\ d & 0 & c \end{pmatrix} \text{ and } \hat{R}(B_g) = \begin{pmatrix} 0 & e & 0 \\ e & 0 & f \\ 0 & f & 0 \end{pmatrix}$$

For parallel configuration used in current study ($\boldsymbol{e_L} \, // \, \boldsymbol{e_s}$), this leads to the following expressions for angular dependencies of the intensities for Raman-active modes

$I^{//}_{A_g}(\varphi) \sim (a \times cos(\varphi)^2 \, + \, b \times sin(\varphi)^2)^2$,

$I^{//}_{B_g}(\varphi) \sim (e \times sin(2\varphi))^2$,

where φ is the angle between the polarization of the excitation and *a* axis (i.e. $\boldsymbol{e_L} = \boldsymbol{e_s} = (cos(\varphi), sin(\varphi), 0)$). We used these dependencies to fit polar diagrams derived from the experimental data.

In case of cross-configuration we have $\boldsymbol{e_L} = (cos(\varphi), sin(\varphi), 0)$, $\boldsymbol{e_L} = (-sin(\varphi), cos(\varphi), 0)$. Therefore, intensities are given as:

$I^{\perp}_{A_g}(\varphi) \sim (\frac{b-a}{2} \times sin(2\varphi))^2$,

$I^{\perp}_{B_g}(\varphi) \sim (e \times cos(2\varphi))^2$.

These equations explain why intensities are close to zero in cross-polarization configuration compared to parallel configuration. First of all, due to the fact that the diagonal components *b* and *a* of the A_g modes are much larger than the non-diagonal *e* components of the B_g modes, B_g modes are weak in the parallel configuration. Secondly, due to the fact that *b* and *a* are of the same order for most of A_g modes, then we see a strong decrease in Ag modes intensity in the cross-polarization. The latest point explains why the most intensive peak A_g^6 (experimental position 360 cm^{-1}) is almost nullified in cross-case. As its polar plot is circle-shaped, values of $b(A_g^6)$ and $a(A_g^6)$ are close to each other. Therefore, the $I(A_g^6)$ intensity, which is proportional to $(a(A_g^6) - b(A_g^6))^2$, is relatively weak.

As a side note, type of the polar plot is indicative of its origin: for A_g (i.e., for all polar diagrams presented in Figure 2), we observe isotropic (for $a \approx b$), "dumbbell-like" (for $a < b$ or $a > b$) or “eight-like” (for $a << b$ or $a >> b$) plots with maxima and minima oriented towards optical axes, while for B_g (for example, for the lines positioned at 117, 137, 179 cm^{-1}) we elicit "clover-like" dependencies with maxima oriented at 45° to the optical axes. Figure S8 illustrates DFT-derived polar plots, showing a clear distinction between A_g and B_g modes.

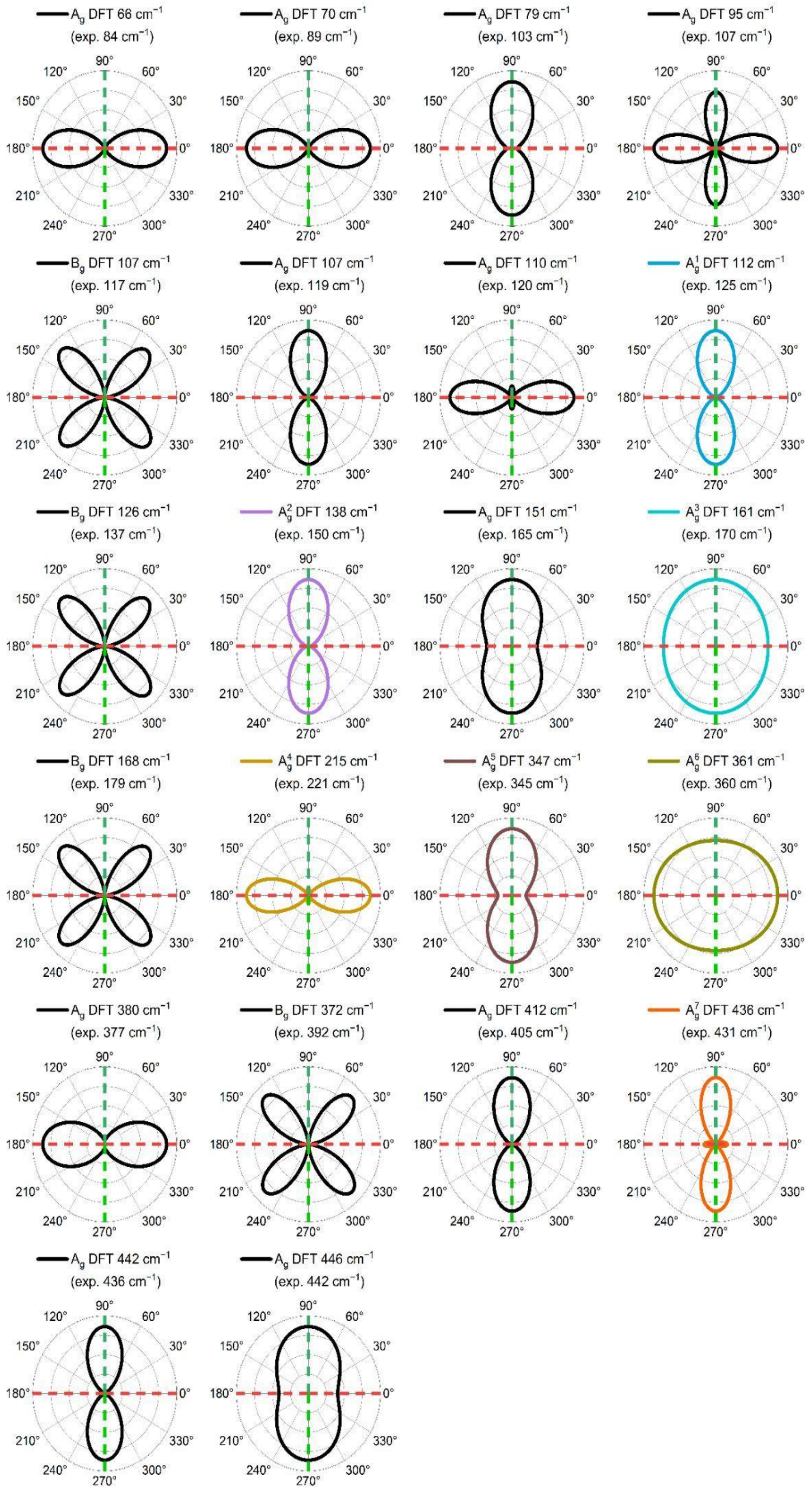

Figure S8. Ab initio polar plots of experimentally observed lines in >100 cm^{-1} range. $A_g^1 - A_g^7$ modes are indicated by colors. Dashed lines are aligned with crystallographic axes *a* (red) and *b* (green).

Non-resonant excitation facilitates only a slight difference between the polar diagrams being derived from the spectra excited by 532 nm (Figure 2) and 633 nm (Figure S9) lasers.

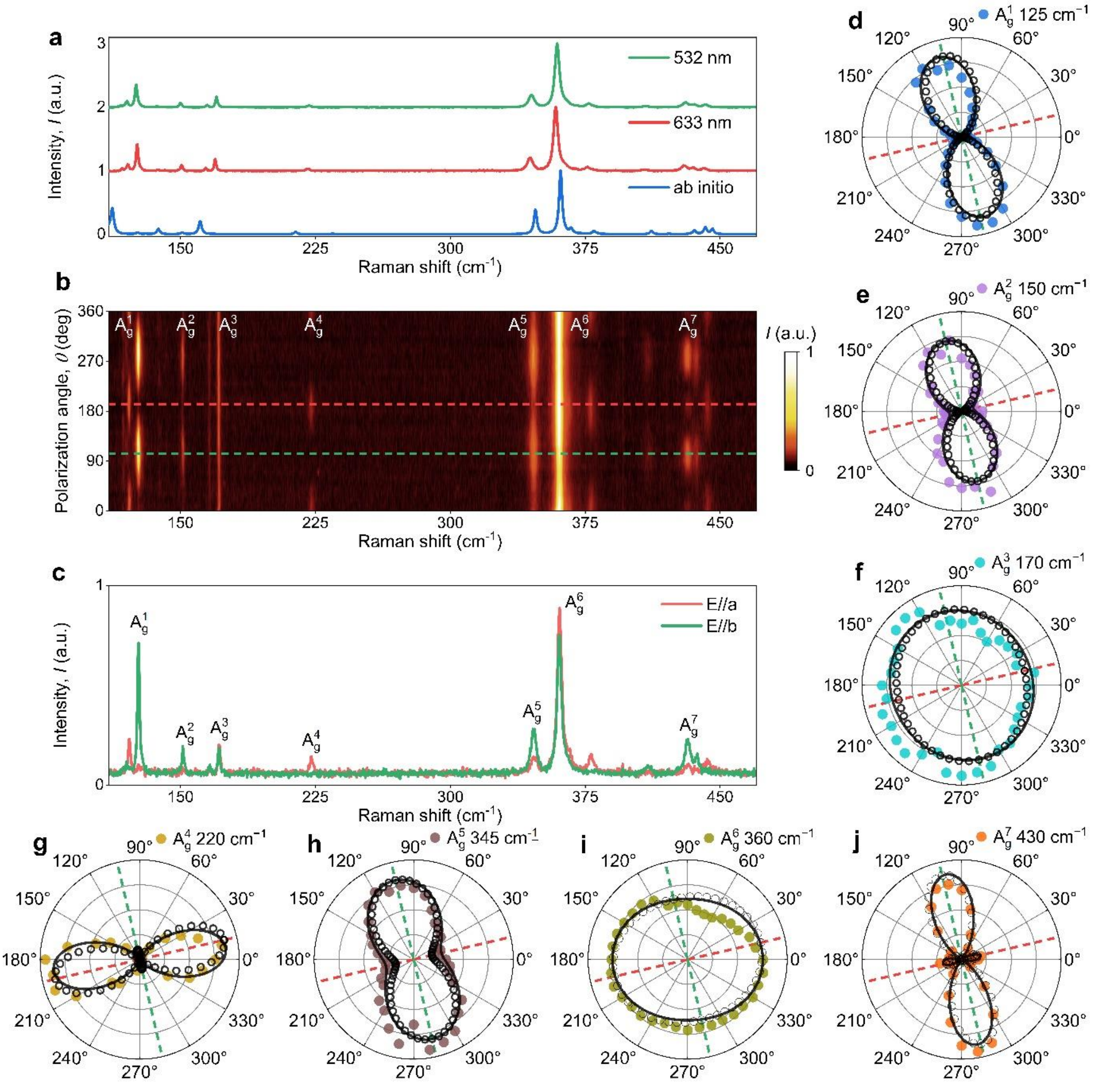

Figure S9. (a) Experimental angle-averaged polarized Raman spectra of GeS_2 measured at excitation wavelengths of 532 nm and 633 nm and calculated. **(b)** Angle-resolved Raman intensity colormap of a bulk GeS_2 flake (thickness, $t > 100$ nm) acquired by rotating a sample in parallel-polarized configuration measured at 633 nm excitation. **(c)** Raman spectra along the two crystalline axes (*a* and *b*) measured at 633 nm excitation. **(d-g)** Polar plots of the fitted peak intensities for 7 Raman modes measured at 633 nm excitation. Solid and dotted black curves indicate normalized calculated polar plots and normalized experimental data fitting respectively. Dashed colored lines indicate crystallographic axes directions: *a* (red) and *b* (green). Experimental data is shown by full circles, its fittings are indicated by solid lines. DFT data is presented by hollow circles.

Next, we attribute selected Raman-active peaks to fundamental vibrations. We describe 13 peaks, and assign A_g^1-A_g^7 notations to 7 of them. In the literature there is a description of only 3 peaks[17], which in our notation correspond to A_g^1 (125 cm^{-1}), A_g^6 (360 cm^{-1}) and unnumbered A_g at 377 cm^{-1}.
We present a brief attribution of the selected peaks in Table S4 along with their visualization placed in Table S5 and Table S6 for numbered and unnumbered peaks respectively. We will focus only on the

description of A_g modes, since both in the experiment and in the calculations the B_g modes have very weak intensities compared to A_g (see Figure S7).
Here we turn our attention to the most intense A_g^6 mode. It has a circle-like polar diagram in parallel configuration and the highest intensity among all the lines. It is explained by its origin, synphase breathing of all GeS_4 tetrahedra, which ensues intensive and isotropic light scattering. Our attribution differs from that described in [17], where, based on valence force field (VFF) method, the authors claimed that only corner-sharing tetrahedra breathe, while the others tetrahedra reportedly “do not vibrate much, though they move rotationally or translationally”.
As for other peaks described in[17], such as A_g^1 (125 cm^{-1}) and unnumbered A_g at 377 cm^{-1}, our vibrational assignment doesn't contain any considerable differences from the one obtained by VFF-based approach. We discuss the origins of these lines among other peaks in Table S5 and depict their vibrational pattern based on ab initio calculation in Table S6.
Therefore, our first-principle calculations of phonons clarify the origin of the peaks, which was previously analyzed by the VFF approach, as DFT provides considerably more information on the atomic vibrations than the empirical VFF method. In addition, ab initio approach makes it possible to provide an accurate visualization of vibrational modes.
Next, we discuss the origin of 10 more lines whose vibrational modes haven’t been reported in the literature previously. One interesting point is related to three close distinct experimental peaks in the vicinity of 440 cm^{-1}: A_g^7 (431 cm^{-1}) and two unnumbered A_g bands at 436 and 442 cm^{-1} have very similar vibrational behavior. Their positions are relatively close, as these peaks originate from the stretching of edges consisting of two edge S-atoms. Also, symmetrical stretching of S−Ge−S bonds within the corner-sharing chains contributes to the vibrations at 430 (A_g^7) and 443 cm^{-1}. In the 443 cm^{-1} mode, the edge pattern stretches synchronously with the stretching of S−Ge−S bonds. In the A_g^7 mode it’s the other way around: the edge pattern is stretched synchronously with the compression of S−Ge−S bonds. The other peaks are attributed, as mentioned above, in Table S4.

Table S4. Brief assignment of selected Raman-active peaks. **Irrep** – irreducible representation of a given mode, ν_{exp} and ν_{DFT} – experimental (using 532 nm laser) and DFT Raman shifts respectively.

**Irrep**	**ν_{exp}, cm^{-1}**	**ν_{DFT}, cm^{-1}**	**Attribution**
A_g^1	125	112.0	Twisting of corner S-atoms in S−Ge−S corner-sharing chains and rotation of $(GeS)_2$ rings.
A_g^2	150	137.6	Synchronized scissoring of S−Ge−S bonds with S-bridge atoms and Ge-ring atoms. “Synchronized” means that different scissors open and close in the same phase. Corner-sharing units and rings are practically motionless compared to the scissoring.
A_g^3	170	160.9	Libration of $(GeS)_2$ rings and twisting of Ge atoms in Ge-S-Ge corner-sharing chains.

A_g^4	220	213.9	In-phase breathing of $(GeS)_2$ rings.
A_g^5	345	347.3	Low-amplitude breathing of corner-sharing tetrahedra together with high-amplitude asymmetric stretching of S–Ge–S bonds inside rings $(GeS)_2$.
A_g^6	360	361.3	Synphase breathing of all GeS_4 tetrahedra.
A_g^7	430	435.7	Slight symmetric stretching of S–Ge–S bonds in corner-sharing chains and strong stretching of S–S edge in edge-sharing patterns.
Non-indexed A_g modes			
A_g	119	106.6	Prominent wagging of corner-S atoms in S–Ge–S chains. Vertical (out-of-plane) oscillations of $(GeS)_2$ rings as rigid units. Slight oscillations of bridge-S atoms.
A_g	120	110.1	Mixed rotation and libration of $(GeS)_2$ rings. Twisting of Ge atoms in Ge–S–Ge corner-sharing chains. Noticeable oscillations of bridging S atoms.
A_g	377	379.7	Antisymmetric stretching of the following bonds: 1) Ge–S–Ge in chains of corner-sharing tetrahedra, 2) Ge–S–Ge with bridge-sulfur atoms connecting germanium atoms in ring and chain, 3) S–Ge–S with two bridge-sulfur atoms and ring germanium.
A_g	409	411.8	Asymmetric stretching of S–Ge–S bonds with bridging S-atoms in all of the GeS_4 tetrahedra. Despite the fact that the stretching inside one tetrahedron is asymmetric, the collective movement is arranged in such a way that when passing within one chain of “corners” from a tetrahedron to a tetrahedron, an asymmetric oscillation of the type S⟶Ge⟶S changes to S⟵Ge⟵S, which explains the Raman activity of this mode.
A_g	436	441.8	Strong stretching of S–S edge in edge-sharing patterns.
A_g	442	445.7	Simultaneous stretching of S–S edge in edge-sharing patterns and symmetric stretching of S–Ge–S bonds with corner-S atoms. According to our findings, this mode is relatively low-intensive in comparison to the second order of 220 cm^{-1} A_g: see the discussion below.

Table S5. $A_g^1 - A_g^7$ modes visualization.

A_g^1	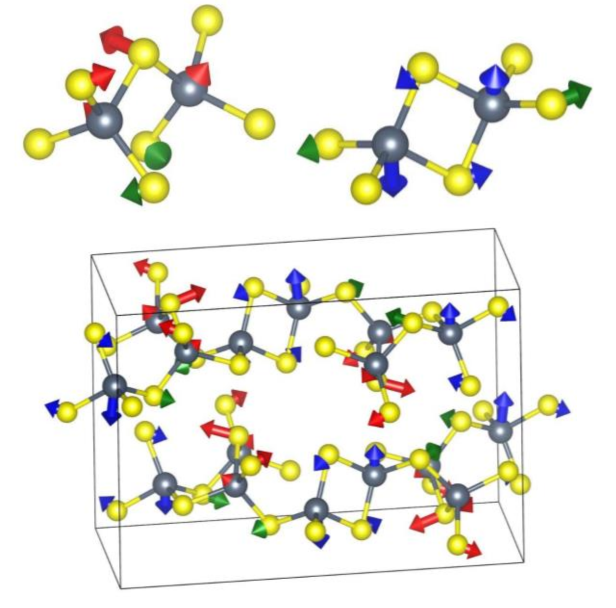	A_g^2	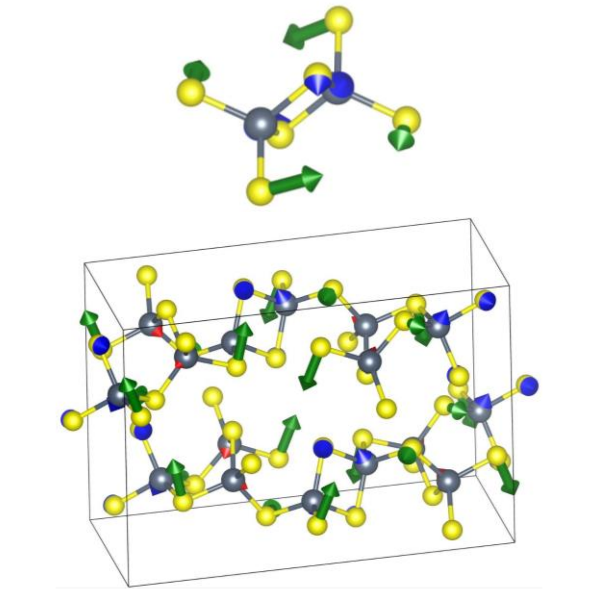
A_g^3	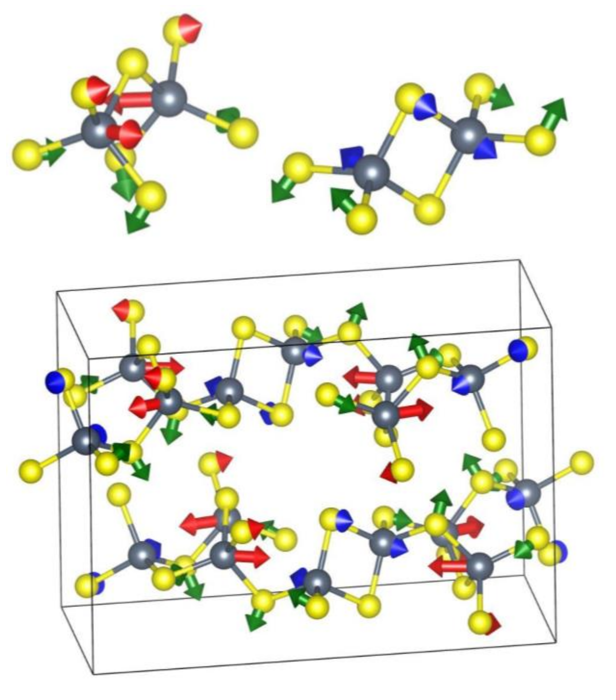	A_g^4	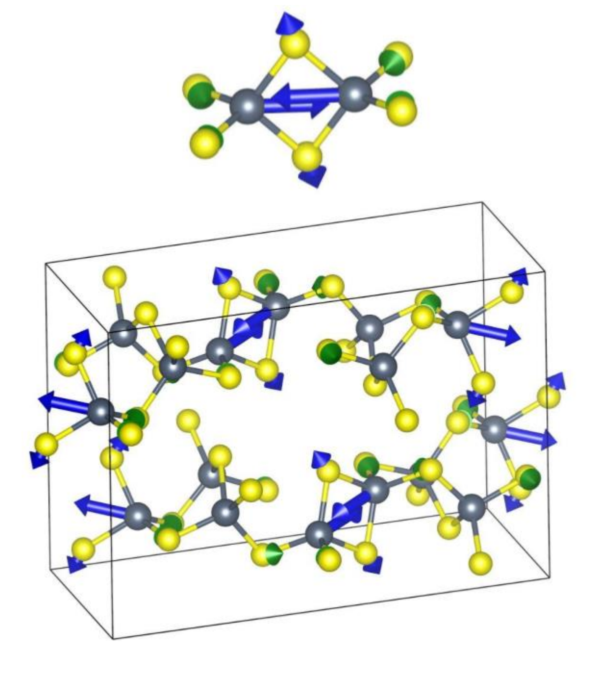

A_g^5	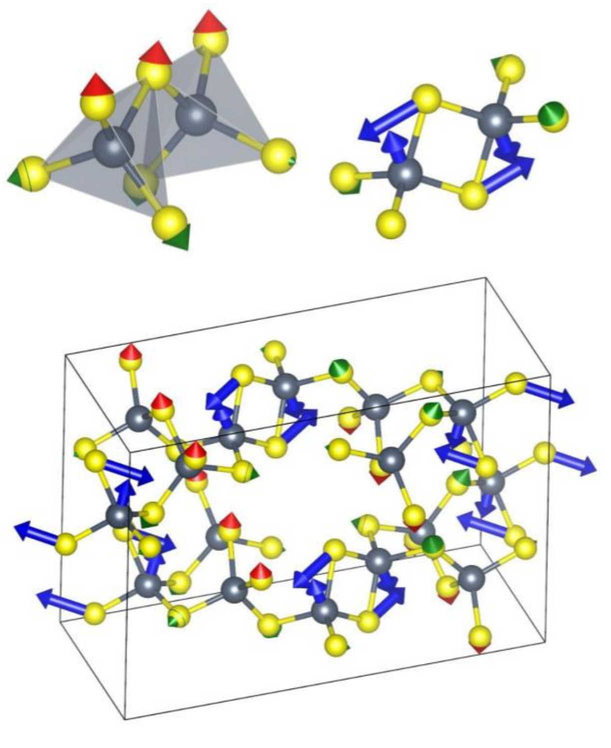	A_g^6	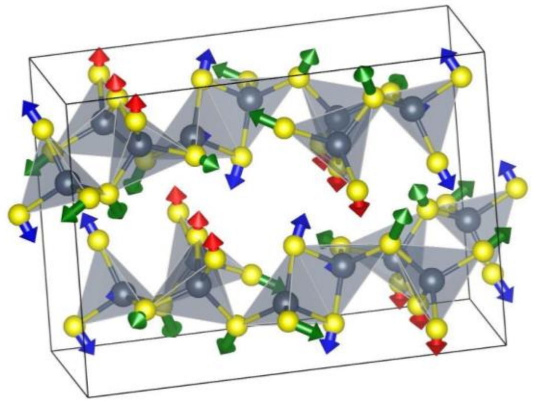
A_g^7	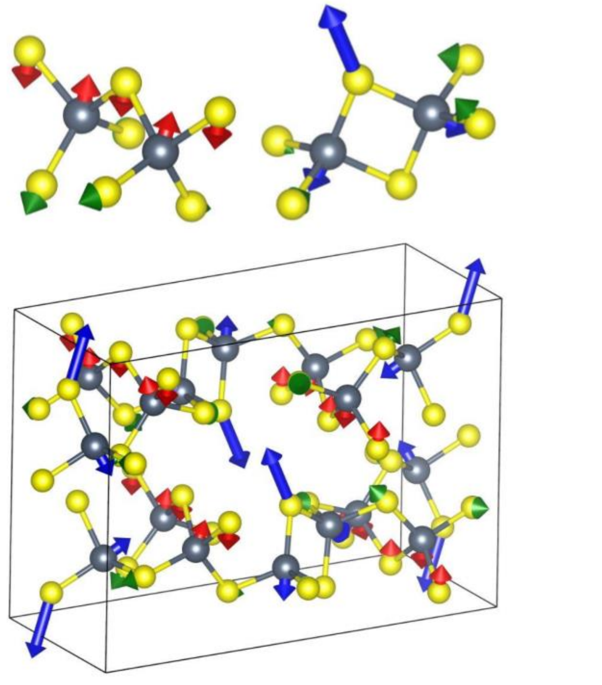		

Table S6. Visualization of the selected non-indexed A_g modes. Two float numbers identifying each mode are Raman shifts obtained experimentally and by DFT calculations respectively.

119, 106.6 cm^{-1}	120, 110.1 cm^{-1}
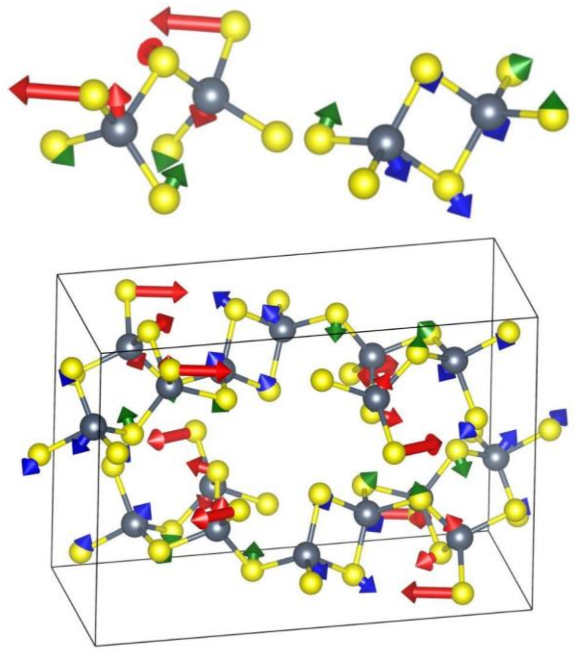	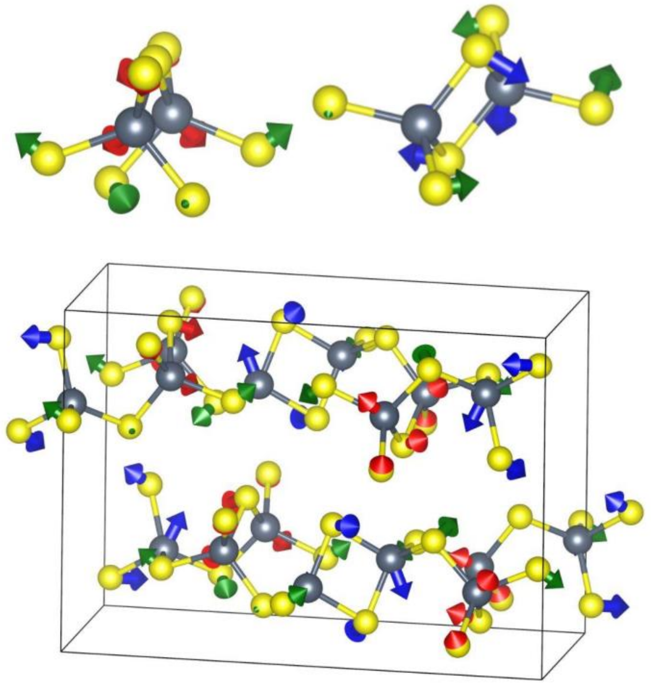
377, 379.7 cm^{-1}	409, 411.8 cm^{-1}
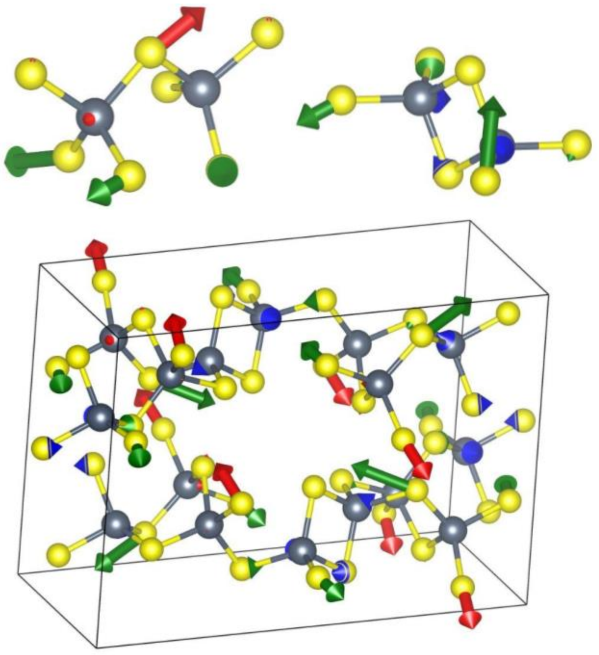	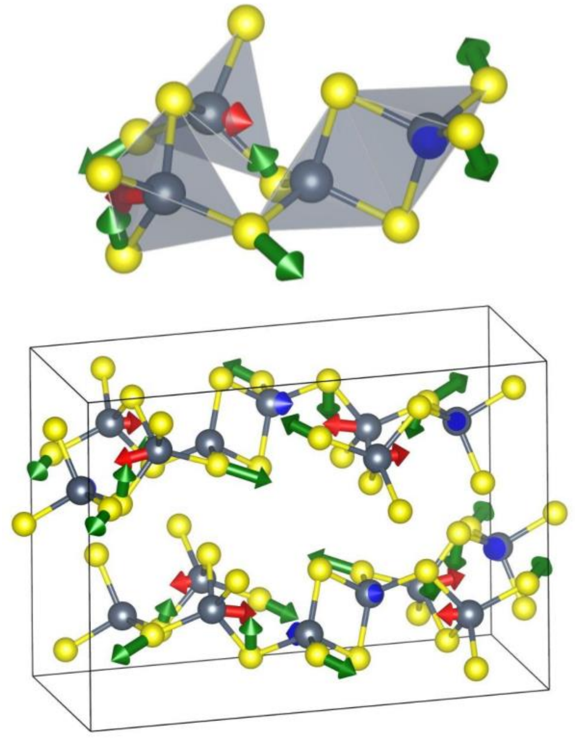
434, 441.8 cm^{-1}	442, 445.7 cm^{-1}

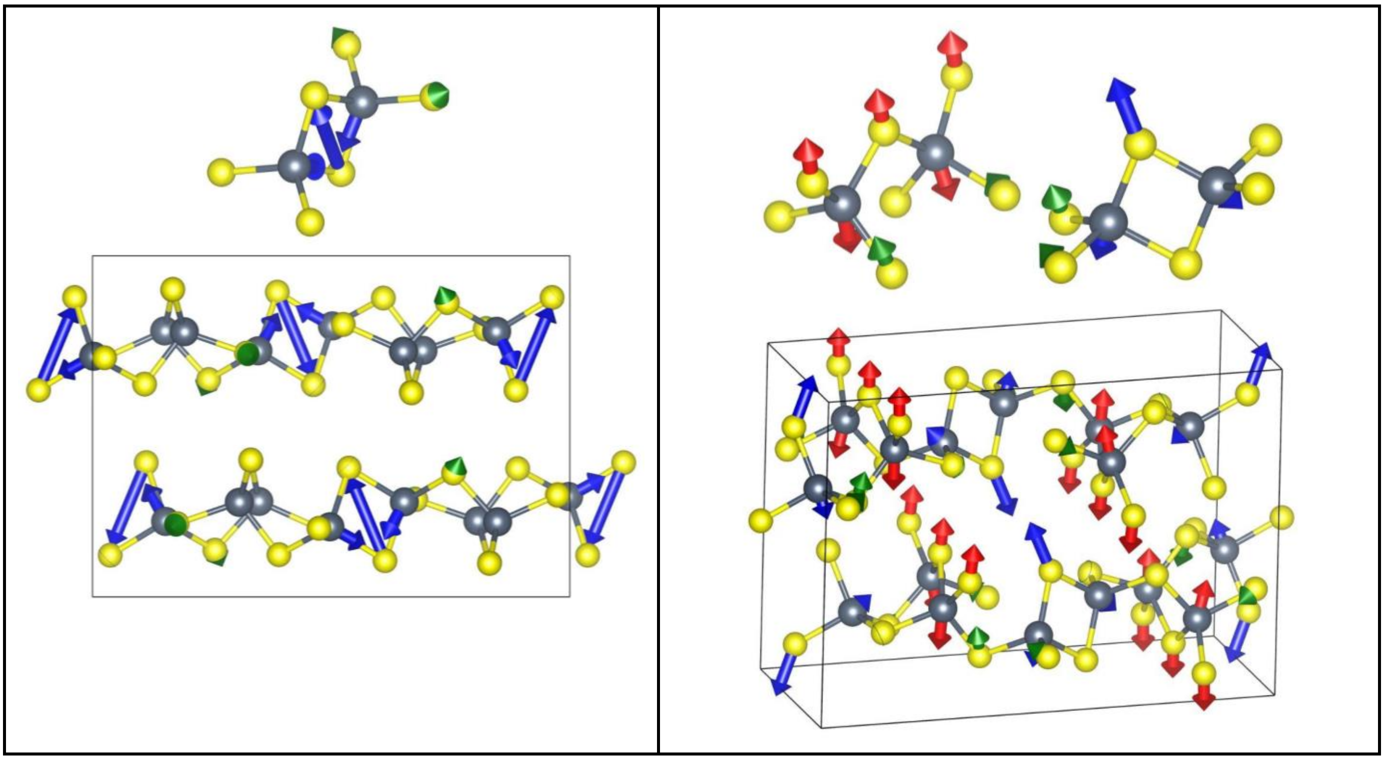

Figure S10 and Figure S11 show angle-resolved intensities measured at 532 nm and 633 nm in $\boldsymbol{e_s}$ // $\boldsymbol{e_L}$ configuration, respectively and *ab initio* calculated polar plots. In these plots, 0° is aligned to be parallel to the crystallographic axis a. 633-nm-excited spectra allowed us to resolve a low-wavenumber 84-cm^{-1}-centered line due to the limit of the edge-filter being lower than the one of the 533 nm edge-filter. However, due to low intensity of 633-nm-excited spectra, we haven't resolved 179 cm^{-1} and 392 cm^{-1} B_g modes being clearly observable at 532 nm excitation. Noteworthy, in previous angle-resolved Raman studies of 2D-GeS_2, the polarization dependence was observed for the most intensive line only (360 cm^{-1})[18]. As we discussed in the previous section, this line is attributed to the synphase breathing of all GeS_4 tetrahedra, which results in a relatively isotropic ($a \approx b$) polarization dependence of this line making it unsuitable for the crystallographic axes determination. Thus, identification of 21 new angular dependencies can provide new degrees of freedom in the characterization of the devices containing monoclinic layered GeS_2 as a component. For all lines except 442 cm^{-1}, ab initio calculations corroborate well with the experimentally-derived dependencies. The discrepancy between ab initio and experimental polar plots of 442 cm^{-1} line can be explained as observation of overtone, i.e. second-order Raman scattering, in the experiment. Since A_g^4 mode at 220 cm^{-1} has an A_g irreducible representation, we expect it to give a second harmonic at ~2×220 cm^{-1} to be A_g mode as well, since the direct product of two A_g modes is also an A_g mode, according to symmetry theory. We are unable to capture this feature in the theoretical method used, since it is aimed to simulate first-order Raman peaks only. Thus, we suggest a second order of A_g^4 220 cm^{-1} overlaps the ~440-cm^{-1}-centered A_g peak.

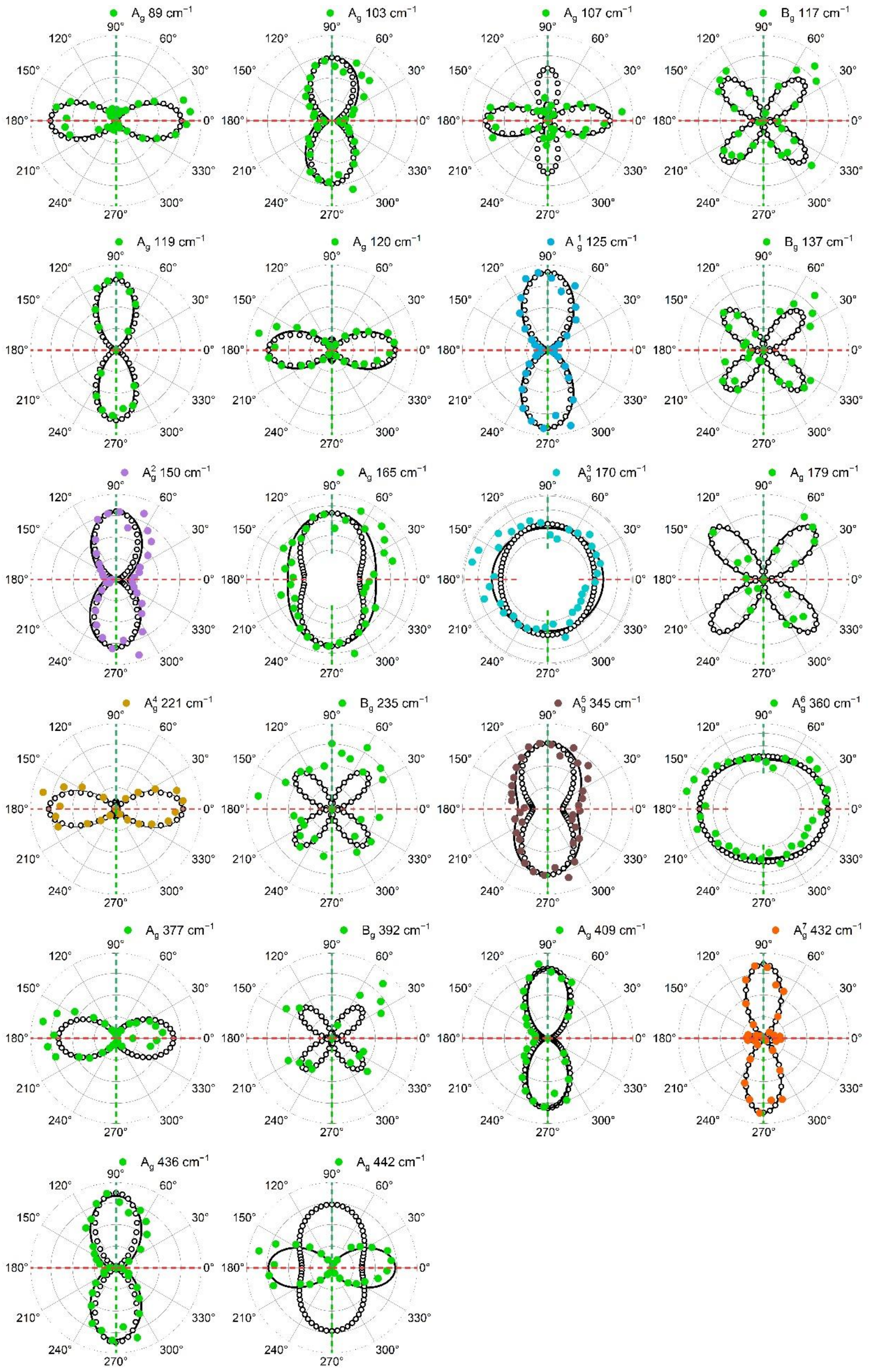

Figure S10. Polarization dependence of the normalized Raman intensities excited at 532 nm. Experimental data is shown by full circles, its fittings are indicated by solid lines. DFT data is presented by hollow circles. $A_g^1 - A_g^7$ modes are indicated by colors. Dashed lines are aligned with crystallographic axes *a* (red) and *b* (green).

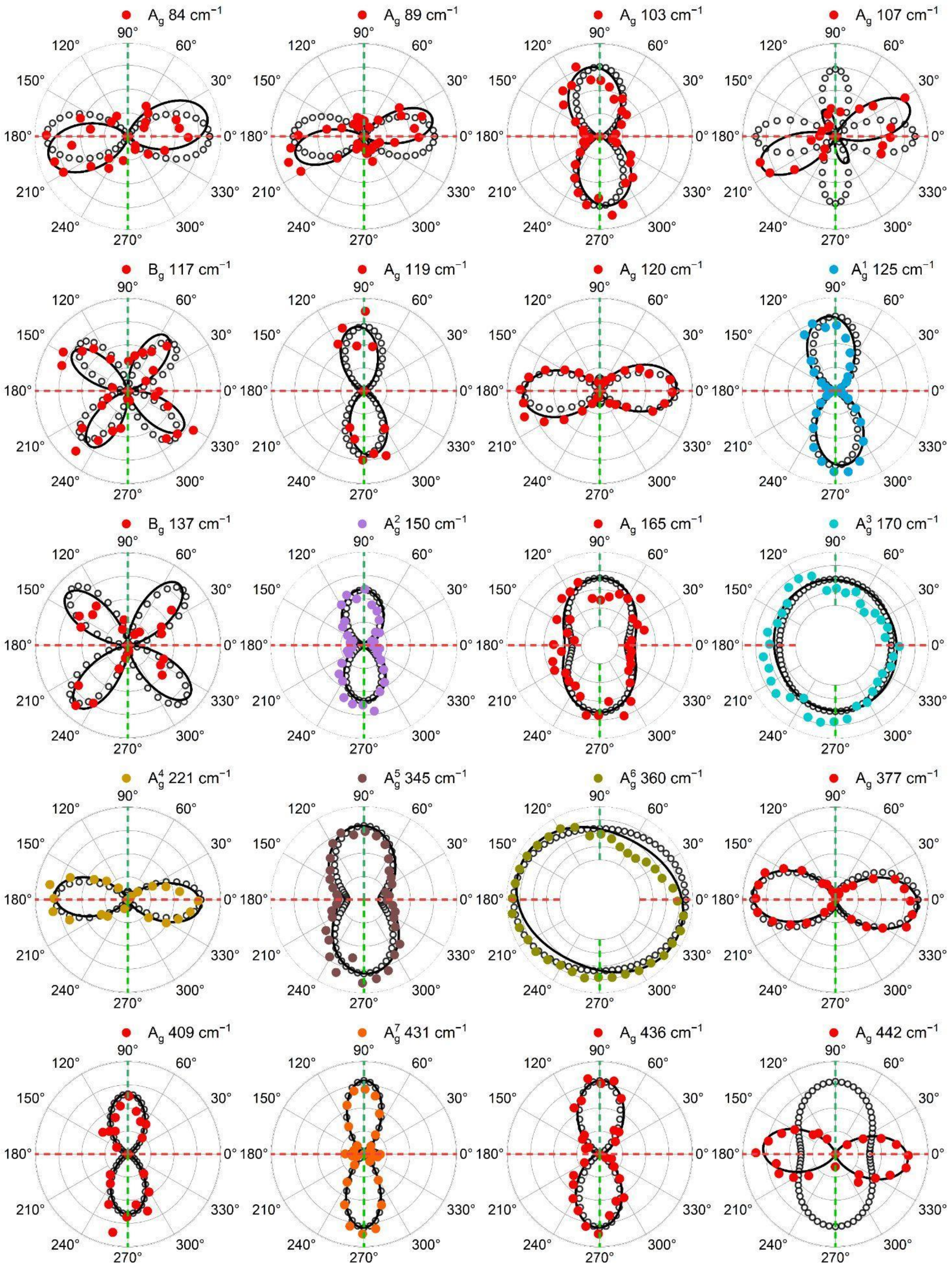

Figure S11. Polarization dependence of the normalized Raman intensities excited at 633 nm. Experimental data is shown by full circles, its fittings are indicated by solid lines. DFT data is presented by hollow circles. $A_g^1 - A_g^7$ modes are indicated by colors. Dashed lines are aligned with crystallographic axes *a* (red) and *b* (green).

On the whole, analysis of angle-resolved Raman response allowed us to elaborate on the origin of three previously-ascribed peaks (A_g^1 (125 cm^{-1}), A_g^6 (360 cm^{-1}) and A_g^i at 377 cm^{-1}) and provide an insight into the origin of the vibrations of 10 more relatively intensive lines (see Table S4). Among these lines, we have chosen 7 well-resolved intensive modes ($A_g^1 - A_g^7$), whose presence can reliably identify monoclinic layered GeS_2 phase. Noteworthy, A_g^1, A_g^2, A_g^4, A_g^5, A_g^7 peaks have prominently anisotropic intensity with maximums and minimums aligned with a and c crystallographic axes, as confirmed by DFT calculations, thus allowing a reliable identification of the GeS_2 flakes orientation.

Supplementary Note 4: Ellipsometry analysis for determination of GeS_2 optical constants

To obtain the dielectric tensor we performed spectroscopic ellipsometry measurements of GeS_2 flakes on Si substrates. Figure S12 and Figure S13 represent Ψ, Δ spectra at different incidence angles (45°, 50°, 55°).

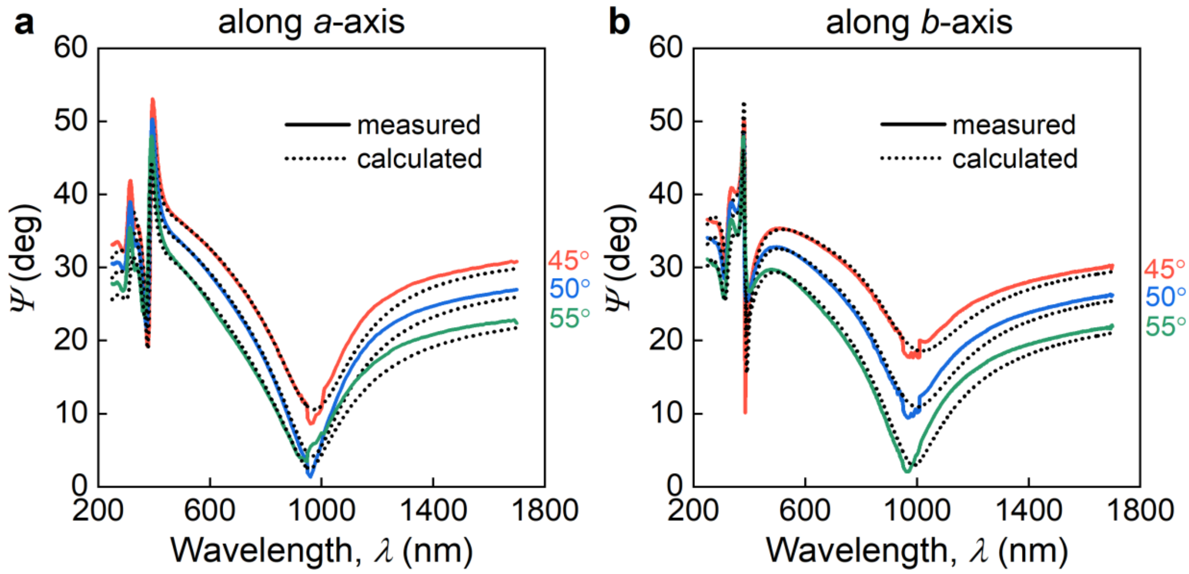

Figure S12. Experimental and simulated ellipsometry amplitude Ψ along crystallographic (a) *a*-axis and (b) *b*-axis.

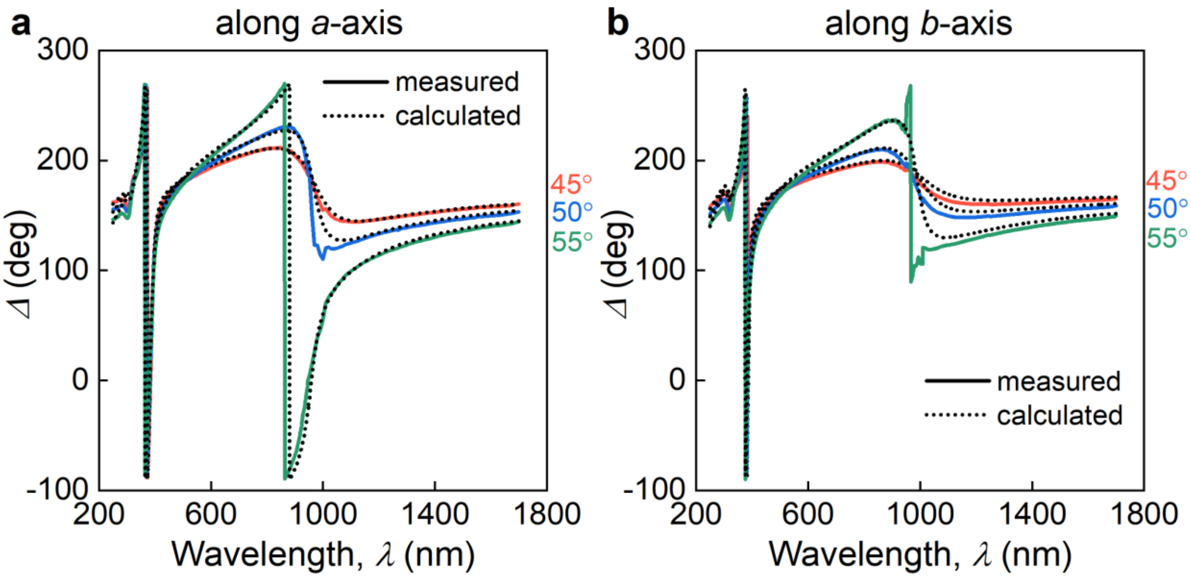

Figure S13. Experimental and simulated ellipsometry amplitude Δ along crystallographic (a) *a*-axis and (b) *b*-axis.

Supplementary Note 5: Processing of polarization-dependent transmittance spectra for GeS_2

To elucidate the orientation of the principal optical axes relative to the crystallographic axes via polarized transmittance measurements, we exfoliated a flake of GeS_2. We then proceeded with micro-transmission measurements under parallel-polarized setup (detailed in Methods). With the polarizer and analyzer fixed, the sample was rotated by 360° in 5° step, capturing the transmittance spectrum within the 500-900 nm wavelength range at each angle. Figure S14 illustrates the resulting color map of transmittance spectra corresponding to all rotation angles. Subsequently, the experimental spectra (Figure S14) were linearly approximated over 1 nm intervals ($\Delta\lambda$) and fitted them as follows:

$$T(\theta) = (E/E_0)^2 = \left|a(cos\ \theta - \theta_0\ )^2 + b(sin\ \theta - \theta_0\ )^2 e^{i\Delta\varphi}\right|^2 =$$

$$= a^2(cos\ \theta - \theta_0)^4 + b^2(sin\ \theta - \theta_0\ )^4 + 2ab(cos\ \theta - \theta_0)^2(sin\ \theta - \theta_0)^2 cos\ \Delta\varphi$$

where $T(\theta)$ is the transmittance at a given wavelength λ and a polarization angle θ; $\Delta\varphi$ is the phase retardance, θ_0 and $\theta_0 + 90°$ represents the positions of principal axes. We used the fitted curves to experimentally detect in-plane principal optical axes orientation.

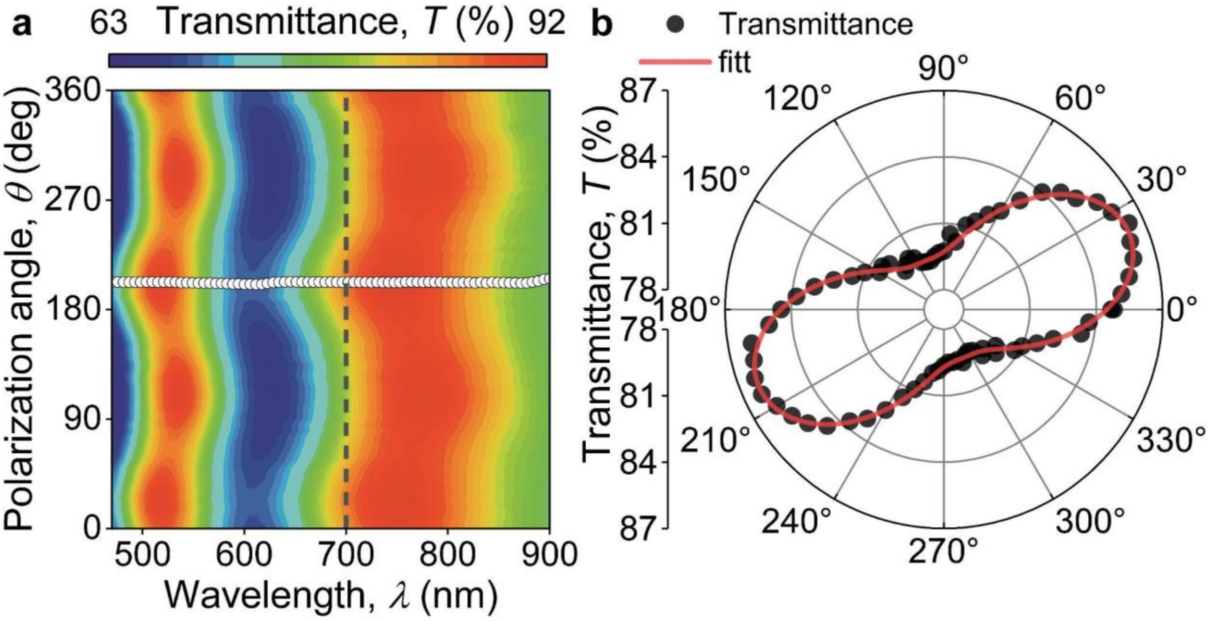

Figure S14. (a) Experimental polarized micro-transmittance map of 334-nm-thick GeS_2 flake. **(b)** Representative angle-dependent transmittance diagram taken along the dashed line in the panel **a**.

Supplementary Note 6: Scanning near-field optical microscopy of GeS_2

To extract the effective indices of the modes propagating in the GeS_2 planar waveguide, we used third harmonics o3A and o3P, as shown in Figure S15a-c and Figure S15d-f respectively. The difference in the frequency of the observed oscillations near the top and right edges is not so much associated with the anisotropy of the material, but rather with a frequency shift due to the geometry of the experiment, as described in the main text.

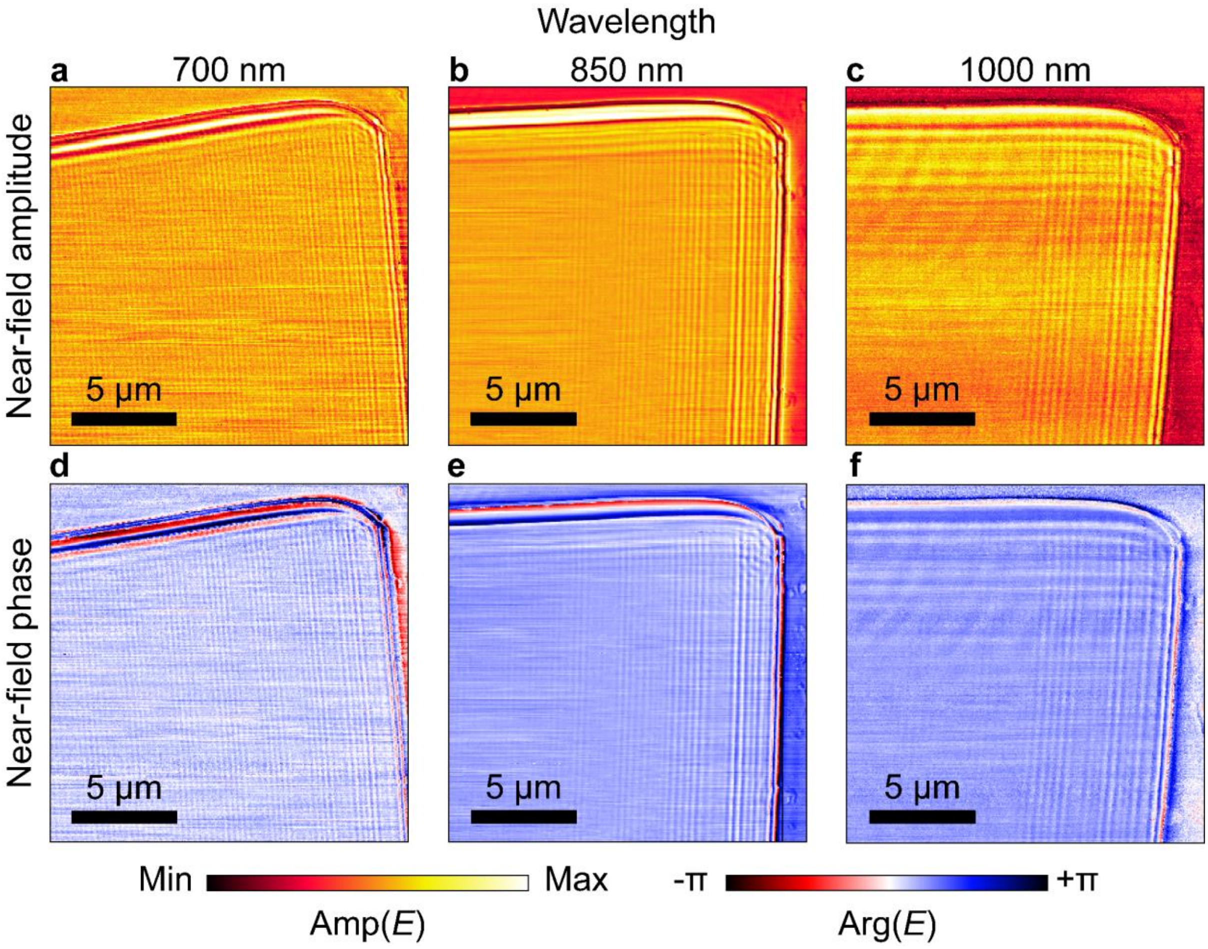

Figure S15. Near-field images: **(a-c)** amplitude *Amp(E)* at incident wavelengths 700 nm; 850 nm; 1000 nm; (**d-f)** phase *Arg(E)* at incident wavelengths 700 nm; 850 nm; 1000 nm.

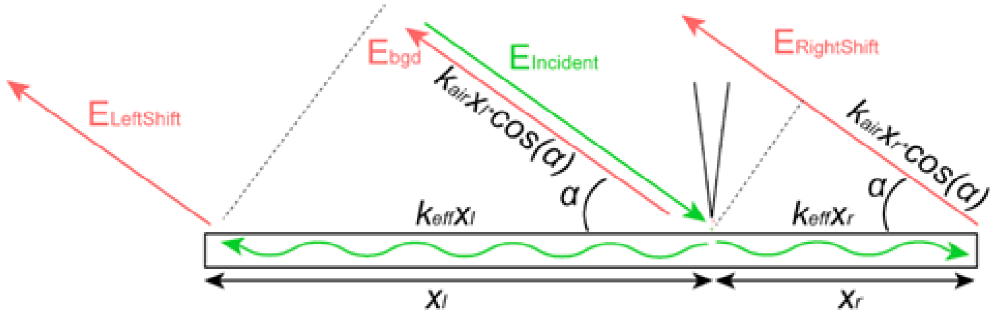

Figure S16. Schematic 2D illustration of the frequency shift effect in s-SNOM measurements.

Figure S16 represents a 2D schematic illustration of frequency shift effect in scattering-type SNOM. The effect appears due to the interference between the background field E_{bgd} and the field scattered

from the edge of the sample ($E_{RightShift}$ or $E_{LeftShift}$). It can be clearly seen from the scheme that the phase difference in those fields depends on the distance to the edge of the sample, where scattering occurs. Thus, it leads to the frequency shift effect in the observed effective mode indices.

Supplementary Note 7: Tabulated optical constants of GeS_2

Table S7. Tabulated optical constants of GeS_2.

λ (nm)	n_a (in-plane)	k_a (in-plane)	n_b (in-plane)	k_b (in-plane)	n_c (out-of-plane)	k_c (out-of-plane)
250	1.98321	1.22553	1.9043	1.45366	2.32937	0
260	2.21787	1.29581	2.2064	1.59374	2.29759	0
270	2.49427	1.28489	2.59969	1.61841	2.26927	0
280	2.76596	1.16237	3.00517	1.44979	2.24394	0
290	2.96369	0.94041	3.27373	1.10748	2.22118	0
300	3.04662	0.68268	3.33835	0.73738	2.20065	0
310	3.03205	0.45468	3.26887	0.4513	2.18208	0
320	2.96379	0.28362	3.15181	0.26276	2.16523	0
330	2.87685	0.16651	3.0322	0.14607	2.14988	0
340	2.79002	0.09061	2.92568	0.07584	2.13587	0
350	2.71124	0.04374	2.83552	0.03486	2.12304	0
360	2.64311	0.01683	2.76087	0.01249	2.11126	0
370	2.58608	0.00364	2.70005	0.00224	2.10043	0
380	2.54033	0	2.65209	0	2.09044	0
390	2.50646	0	2.61592	0	2.08121	0
400	2.47967	0	2.58681	0	2.07266	0
410	2.45755	0	2.56259	0	2.06473	0
420	2.43887	0	2.54204	0	2.05736	0
430	2.42284	0	2.52434	0	2.0505	0
440	2.40889	0	2.50892	0	2.0441	0
450	2.39665	0	2.49536	0	2.03813	0
460	2.38581	0	2.48333	0	2.03254	0
470	2.37614	0	2.47259	0	2.0273	0
480	2.36746	0	2.46294	0	2.02238	0
490	2.35963	0	2.45423	0	2.01777	0
500	2.35253	0	2.44633	0	2.01342	0
510	2.34606	0	2.43913	0	2.00933	0
520	2.34015	0	2.43255	0	2.00548	0
530	2.33472	0	2.42651	0	2.00184	0
540	2.32973	0	2.42094	0	1.9984	0
550	2.32512	0	2.41581	0	1.99514	0
560	2.32085	0	2.41105	0	1.99206	0
570	2.31689	0	2.40664	0	1.98914	0
580	2.31321	0	2.40254	0	1.98637	0
590	2.30978	0	2.39871	0	1.98374	0

600	2.30657	0	2.39514	0	1.98124	0
610	2.30357	0	2.39179	0	1.97887	0
620	2.30075	0	2.38866	0	1.9766	0
630	2.29811	0	2.38571	0	1.97444	0
640	2.29563	0	2.38294	0	1.97239	0
650	2.29329	0	2.38033	0	1.97042	0
660	2.29108	0	2.37787	0	1.96855	0
670	2.28899	0	2.37554	0	1.96676	0
680	2.28702	0	2.37335	0	1.96505	0
690	2.28515	0	2.37126	0	1.96341	0
700	2.28338	0	2.36929	0	1.96184	0
710	2.2817	0	2.36741	0	1.96034	0
720	2.2801	0	2.36563	0	1.9589	0
730	2.27858	0	2.36394	0	1.95751	0
740	2.27713	0	2.36233	0	1.95619	0
750	2.27576	0	2.36079	0	1.95491	0
760	2.27444	0	2.35933	0	1.95369	0
770	2.27319	0	2.35793	0	1.95251	0
780	2.27199	0	2.3566	0	1.95138	0
790	2.27084	0	2.35532	0	1.95029	0
800	2.26975	0	2.3541	0	1.94925	0
810	2.2687	0	2.35293	0	1.94824	0
820	2.26769	0	2.35181	0	1.94726	0
830	2.26673	0	2.35073	0	1.94633	0
840	2.2658	0	2.3497	0	1.94542	0
850	2.26491	0	2.34871	0	1.94455	0
860	2.26406	0	2.34776	0	1.94371	0
870	2.26324	0	2.34685	0	1.94289	0
880	2.26245	0	2.34597	0	1.94211	0
890	2.26169	0	2.34513	0	1.94135	0
900	2.26096	0	2.34431	0	1.94061	0
910	2.26026	0	2.34353	0	1.9399	0
920	2.25958	0	2.34277	0	1.93921	0
930	2.25893	0	2.34205	0	1.93855	0
940	2.2583	0	2.34134	0	1.9379	0
950	2.25769	0	2.34066	0	1.93728	0
960	2.2571	0	2.34001	0	1.93667	0
970	2.25653	0	2.33938	0	1.93609	0
980	2.25598	0	2.33876	0	1.93552	0

990	2.25545	0	2.33817	0	1.93497	0
1000	2.25494	0	2.3376	0	1.93444	0
1010	2.25444	0	2.33705	0	1.93392	0
1020	2.25396	0	2.33651	0	1.93341	0
1030	2.2535	0	2.33599	0	1.93292	0
1040	2.25304	0	2.33549	0	1.93245	0
1050	2.25261	0	2.335	0	1.93199	0
1060	2.25218	0	2.33453	0	1.93154	0
1070	2.25177	0	2.33407	0	1.9311	0
1080	2.25137	0	2.33363	0	1.93068	0
1090	2.25099	0	2.3332	0	1.93027	0
1100	2.25061	0	2.33278	0	1.92987	0
1110	2.25025	0	2.33237	0	1.92948	0
1120	2.24989	0	2.33198	0	1.9291	0
1130	2.24955	0	2.3316	0	1.92873	0
1140	2.24921	0	2.33122	0	1.92837	0
1150	2.24889	0	2.33086	0	1.92802	0
1160	2.24857	0	2.33051	0	1.92767	0
1170	2.24826	0	2.33017	0	1.92734	0
1180	2.24796	0	2.32983	0	1.92702	0
1190	2.24767	0	2.32951	0	1.9267	0
1200	2.24739	0	2.32919	0	1.92639	0
1210	2.24711	0	2.32889	0	1.92609	0
1220	2.24684	0	2.32858	0	1.9258	0
1230	2.24658	0	2.32829	0	1.92551	0
1240	2.24633	0	2.32801	0	1.92523	0
1250	2.24608	0	2.32773	0	1.92496	0
1260	2.24583	0	2.32746	0	1.92469	0
1270	2.2456	0	2.3272	0	1.92443	0
1280	2.24537	0	2.32694	0	1.92418	0
1290	2.24514	0	2.32669	0	1.92393	0
1300	2.24492	0	2.32645	0	1.92369	0
1310	2.24471	0	2.32621	0	1.92345	0
1320	2.2445	0	2.32597	0	1.92322	0
1330	2.24429	0	2.32575	0	1.92299	0
1340	2.2441	0	2.32552	0	1.92277	0
1350	2.2439	0	2.32531	0	1.92255	0
1360	2.24371	0	2.3251	0	1.92234	0
1370	2.24352	0	2.32489	0	1.92213	0

1380	2.24334	0	2.32469	0	1.92193	0
1390	2.24316	0	2.32449	0	1.92173	0
1400	2.24299	0	2.3243	0	1.92154	0
1410	2.24282	0	2.32411	0	1.92135	0
1420	2.24265	0	2.32392	0	1.92116	0
1430	2.24249	0	2.32374	0	1.92098	0
1440	2.24233	0	2.32356	0	1.9208	0
1450	2.24218	0	2.32339	0	1.92063	0
1460	2.24202	0	2.32322	0	1.92046	0
1470	2.24188	0	2.32306	0	1.92029	0
1480	2.24173	0	2.32289	0	1.92013	0
1490	2.24159	0	2.32273	0	1.91997	0
1500	2.24145	0	2.32258	0	1.91981	0
1510	2.24131	0	2.32243	0	1.91965	0
1520	2.24118	0	2.32228	0	1.9195	0
1530	2.24105	0	2.32213	0	1.91935	0
1540	2.24092	0	2.32199	0	1.91921	0
1550	2.24079	0	2.32185	0	1.91907	0
1560	2.24067	0	2.32171	0	1.91893	0
1570	2.24055	0	2.32157	0	1.91879	0
1580	2.24043	0	2.32144	0	1.91865	0
1590	2.24031	0	2.32131	0	1.91852	0
1600	2.24019	0	2.32118	0	1.91839	0
1610	2.24008	0	2.32106	0	1.91826	0
1620	2.23997	0	2.32094	0	1.91814	0
1630	2.23987	0	2.32082	0	1.91802	0
1640	2.23976	0	2.3207	0	1.9179	0
1650	2.23966	0	2.32058	0	1.91778	0
1660	2.23955	0	2.32047	0	1.91766	0
1670	2.23945	0	2.32036	0	1.91755	0
1680	2.23936	0	2.32025	0	1.91744	0
1690	2.23926	0	2.32014	0	1.91732	0
1700	2.23916	0	2.32003	0	1.91722	0

Supplementary Note 8: Polarized transmittance calculations (transfer matrix method)

In this study, incident light polarized by the initial polarizer (P_1) is directed perpendicularly onto a GeS_2 flake positioned on a glass substrate (see Figure S17). The transmitted light subsequently passes through the analyzing polarizer (P_2). We examine the dependence of light intensity on the relative orientation of the sample and polarizers P_1 and P_2. For the theoretical determination of the transmission coefficient, we solve Maxwell's equations with the appropriate boundary conditions.

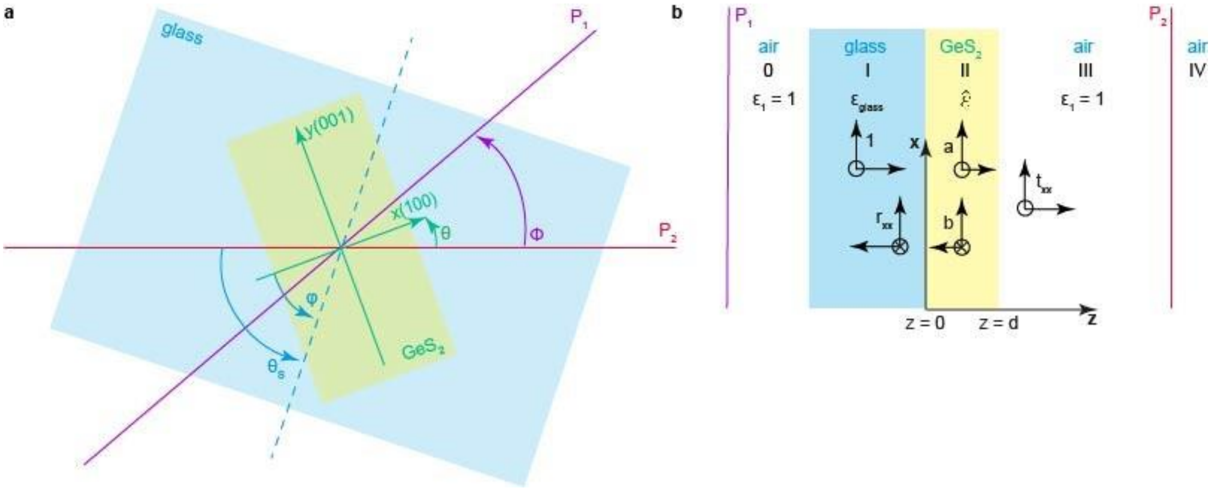

Figure S17. A schematic representation of the experimental setup from top and side perspectives. **(a)** The angular arrangement of the GeS_2 plate, substrate, and polarizers P_1 and P_2 is depicted. **(b)** The field distribution is illustrated, indicating the in-plane electric field components (***E***), represented by arrows, and the out-of-plane magnetic field components (***H***), represented by circles with or without '*x*' depending on their direction.

Initially, we determined the electric field in regions 0 through IV (see Figure S17b). Assuming negligible back-reflections in the glass plate, the following relationship can be established:

$$\mathbf{E}_{\mathrm{III}} = \hat{T}\mathbf{E}_{\mathrm{I}} = \hat{T} t_{\mathrm{air-glass}} \mathbf{E}_0, \tag{8.1}$$

Here, $\hat{T}$ represents the Jones matrix characterizing the transmission through the GeS_2 slab.
In the reference frame of the principal directions of the GeS_2 dielectric permittivity tensor, the Jones vector describing the incident light is expressed as:

$$\mathbf{E}_0 = \begin{pmatrix} \cos(\phi - \theta) \\ \sin(\phi - \theta) \end{pmatrix}, \tag{8.2}$$

Here, θ denotes the angle between the *x*-axis of GeS_2 and the orientation of analyzer P_2, while ϕ represents the angle between P_1 and P_2.
Given the diagonal nature of the matrix $\hat{T}$, equation (8.1) can be reformulated as:

$$\mathbf{E}_{\mathrm{III}} = t_{\mathrm{air-glass}} \begin{pmatrix} t_{xx} \cos(\phi - \theta) \\ t_{yy} \sin(\phi - \theta) \end{pmatrix}. \tag{8.3}$$

To determine the components of the transmission matrix, we model the GeS_2 slab as a Fabry–Pérot resonator:

$$t_{xx} = t^{x}_{\mathrm{I-II}} \Phi t^{x}_{\mathrm{II-III}} + t^{x}_{\mathrm{I-II}} \Phi r^{x}_{\mathrm{II-III}} \Phi r^{x}_{\mathrm{II-I}} \Phi t^{x}_{\mathrm{II-III}} + (\dots) \tag{8.4}$$

Here, $t_{i-j} = 2n_i/(n_i + n_j)$, and $r_{i-j} = t_{i-j} - 1$ represent the transmission and reflection amplitudes at the interface between regions *i* and *j* $(i, j = \mathrm{I}, \mathrm{II}, \mathrm{III})$. The term $\Phi = \exp(ik_x d)$ is the phase accumulation factor, indicating the phase shift a wave with wavenumber k_x undergoes while propagating through the slab of thickness *d*. The superscript *x* on the reflection and transmission amplitudes signifies that for their calculation, we assume $n_{II} = n_x$.
The summation of the infinite geometric series yields

$$t_{xx} = \frac{t^x_{\mathrm{I-II}} t^x_{\mathrm{II-III}} \exp(ik_x d)}{1 - r^x_{\mathrm{II-I}} r^x_{\mathrm{II-III}} \exp(2ik_x d)}. \tag{8.5}$$

A corresponding equation, with appropriate substitutions, applies to t_{yy}.
The electric field amplitude of the detected wave after passing through analyzer P_2 is obtained by projecting the Jones vector in the direction of the analyzer:

$$E_{\mathrm{IV}} = t_{\mathrm{air-glass}}\big(t_{xx}\cos(\phi - \theta)\cos\theta - t_{yy}\sin(\phi - \theta)\sin\theta\big). \tag{8.6}$$

The transmission coefficient of the system is then expressed as:

$$T = \frac{|\mathbf{E}_{\mathrm{IV}}|^2}{|\mathbf{E}_0|^2} = |E_{\mathrm{IV}}|^2 \tag{8.7}$$

It is important to note that the spectrophotometer measures the ratio of the transmission through our system to the transmission through a reference system consisting only of the glass slab. Thus, the value from eq. (8.7) should be adjusted by dividing by $|t_{\mathrm{air-glass}} t_{\mathrm{glass-air}}|^2$:

$$T_{\mathrm{exp}} = \frac{T}{|t_{\mathrm{air-glass}} t_{\mathrm{glass-air}}|^2}, \tag{8.8}$$

Additionally, because the orientation of the analyzer in the experimental setup is fixed and unknown, the experimental angles ϕ and θ may differ from the theoretical angles by a constant shift. By fitting the experimental data to eq. (8.8), we can determine the actual orientation of the analyzer and subsequently obtain the anisotropic refractive indices n_x and n_y of GeS_2.

SUPPLEMENTARY REFERENCES

[1] G. Dittmar, H. Schäfer, *Acta Crystallogr. B* **1975**, *31*, 2060.
[2] A. Togo, F. Oba, I. Tanaka, *Phys. Rev. B Condens. Matter Mater. Phys.* **2008**, *78*, DOI 10.1103/physrevb.78.134106.
[3] A. Togo, I. Tanaka, *Scr. Mater.* **2015**, *108*, 1.
[4] G. Kresse, J. Hafner, *Phys. Rev. B Condens. Matter* **1993**, *47*, 558.
[5] J. P. Perdew, K. Burke, M. Ernzerhof, *Phys. Rev. Lett.* **1996**, *77*, 3865.
[6] S. Grimme, S. Ehrlich, L. Goerigk, *J. Comput. Chem.* **2011**, *32*, 1456.
[7] P. E. Blöchl, *Phys. Rev. B Condens. Matter* **1994**, *50*, 17953.
[8] G. Kresse, D. Joubert, *Phys. Rev. B Condens. Matter* **1999**, *59*, 1758.
[9] X. Gonze, J. Charlier, D. C. Allan, M. P. Teter, *Phys. Rev. B Condens. Matter* **1994**, *50*, 13035.
[10] X. Gonze, C. Lee, *Phys. Rev. B Condens. Matter* **1997**, *55*, 10355.

[11] R. M. Pick, M. H. Cohen, R. M. Martin, *Phys. Rev. B Condens. Matter* **1970**, *1*, 910.
[12] J. Heyd, G. E. Scuseria, M. Ernzerhof, *J. Chem. Phys.* **2003**, *118*, 8207.
[13] G. Kresse, J. Furthmüller, *Phys. Rev. B Condens. Matter* **1996**, *54*, 11169.
[14] G. Kresse, J. Furthmüller, *Comput. Mater. Sci.* **1996**, *6*, 15.
[15] M. Shishkin, G. Kresse, *Phys. Rev. B Condens. Matter Mater. Phys.* **2006**, *74*, DOI 10.1103/physrevb.74.035101.
[16] M. I. Aroyo, J. M. Perez-Mato, C. Capillas, E. Kroumova, S. Ivantchev, G. Madariaga, A. Kirov, H. Wondratschek, *Zeitschrift für Kristallographie - Crystalline Materials* **2006**, *221*, 15.
[17] K. Inoue, O. Matsuda, K. Murase, *Solid State Commun.* **1991**, *79*, 905.
[18] Y. Yang, S.-C. Liu, X. Wang, Z. Li, Y. Zhang, G. Zhang, D.-J. Xue, J.-S. Hu, *Adv. Funct. Mater.* **2019**, *29*, 1900411.
